# Dexamethasone: a double-edged sword in the treatment of osteoarthritis

**DOI:** 10.1038/s41598-025-96050-2

**Published:** 2025-04-07

**Authors:** Karyna Tarasova, Maria Belen Arteaga, Angkana Kidtiwong, Sinan Gueltekin, Andrea Bileck, Christopher Gerner, Iris Gerner, Florien Jenner

**Affiliations:** 1 VETERM, Equine Surgery Unit, Centre for Equine Health and Research, Department for Small Animals and Horses, University of Veterinary Medicine Vienna, Vienna, Austria; 2 Austrian Cluster for Tissue Regeneration, Vienna, Austria; 3 Department of Analytical Chemistry, Faculty of Chemistry, University of Vienna, Vienna, Austria

**Keywords:** Osteoarthritis, Glucocorticoids, Triamcinolone, Dexamethasone, Senescence, Apoptosis, Osteoarthritis, Cartilage

## Abstract

Glucocorticoids are widely used to manage osteoarthritis (OA) symptoms, but long-term safety concerns exist. This study investigates the therapeutic potential of dexamethasone (DEX) and triamcinolone acetonide (TA) in chondrocytes, evaluating their anti-inflammatory effects and potential detrimental actions. This study evaluated the effects of DEX and TA on the expression of pro-inflammatory genes in inflamed chondrocytes. In addition, the effects of DEX treatment on chondrocytes were analyzed using next-generation sequencing, high-resolution mass spectrometry, proliferation and metabolic rate, wound healing capacity and senescence-associated B-galactosidase assays. A single therapeutic dose of DEX (40nM) effectively reduced the expression of inflammatory genes in chondrocytes, while TA showed no such effect. DEX significantly reduced inflammation but also ECM production in inflamed chondrocytes. At 24 h, DEX treatment led to 168 differentially expressed genes (DEGs) compared to untreated inflamed cells, decreasing to 5 DEGs by 48 h, indicating a rapidly diminishing anti-inflammatory effect. Conversely, the difference between DEX-treated and healthy cells increased over time, from 666 DEGs at 24 h to 1317 DEGs at 48 h. Pathway analysis revealed potential disruptions in cell cycle, mitosis, and ECM homeostasis in DEX-treated cells compared to both healthy and inflamed controls. Interestingly, repeated DEX administration at both a therapeutic (40nM) and a high dose (1µM) induced senescence in healthy cells but not in inflamed cells. In contrast, repeated high-dose DEX reduced apoptosis marker Caspase 3/7 in inflamed but not healthy cells. Despite the transient suppression of inflammation achieved with DEX treatment, the observed decrease in ECM production and induction of senescence in healthy chondrocytes at therapeutic doses, along with apoptosis in inflamed cells at higher doses, underscore the need for caution in its intra-articular administration.

**Supplementary Information:**

The online version contains supplementary material available at 10.1038/s41598-025-96050-2.

## Introduction

Glucocorticoids (GC), the most widely prescribed class of drugs globally, are considered the gold standard intraarticular injection to treat symptomatic osteoarthritis^1–3^. GC inhibit the synthesis and secretion of various inflammatory cytokines, prostaglandins and matrix proteinases, and have been shown to reduce glycosaminoglycan loss^4–7^. However, their safety and efficacy in treating OA remain a topic of debate^8–16^. While their anti-inflammatory properties and corresponding ability to temporarily alleviate OA symptoms are well established, concerns have arisen regarding their association with adverse outcomes, including loss of cartilage volume and thickness and OA progression, and deleterious effects on cell viability, proteoglycan synthesis, cartilage morphology and histology^8–16^. Notably, their effect appears to be influenced by treatment dose and duration, with lower doses yielding beneficial effects on cartilage, whereas higher doses exhibit adverse effects, though the interpretation of GC safety and efficacy data is complicated by differences in ages, species, and dosing of the employed models^8–16^.

Triamcinolone acetonide (TA), an intermediate-acting GC with a biological half-life ranging from 18 to 36 h, is one of the most commonly used intraarticular GC^10^, while the synthetic GC dexamethasone (DEX), characterized by its long-acting nature (biological half-life: 36–54 h), is the most potent member of the GC family, exhibiting approximately 5 times higher anti-inflammatory potency compared to TA^8,17^. DEX has demonstrated not just anti-inflammatory and anti-catabolic effects, including modulation of synovial macrophage polarization toward a M2 phenotype^18^, but also anabolic activity, increasing proteoglycan synthesis and promoting chondrogenic differentiation of progenitor cells, suggesting its potential as a disease-modifying drug^4–6,8,19–23^. However, concerns regarding the safety of intraarticular DEX injection have emerged due to reported dose-dependent adverse effects, ranging from chondrocyte death to cartilage degeneration^24–28^. Indeed, extended and/or high-dose DEX treatment induces irreversible cell cycle blockade and a senescence phenotype^29–32^, which may contribute to long-term degenerative changes. This effect is utilized in cancer therapy protocols, which commonly employ high-dose DEX as an adjuvant treatment in solid tumors, and in research to induce senescence^29,32,33^.

Therefore, this study aims to (1) compare the therapeutic efficacy of DEX and TA in reducing inflammation in articular chondrocytes to determine which treatment is more beneficial; (2) evaluate the effectiveness and safety of a single DEX dose for treating inflamed articular chondrocytes using mRNASeq, miRNASeq and Mass-spectrometry proteomics; and (3) determine the safety of repeated DEX administrations in a therapeutic dose. Due to the higher prevalence of OA in females and the paucity of OA-related research in this more commonly afflicted sex, this study is carried out using female ovine donor chondrocytes.

## Materials and methods

### Isolation and culture (2D and 3D pellets) of primary ovine chondrocytes

Primary ovine articular chondrocytes (*n* = 3 biological replicates), which had been previously isolated and biobanked from female Merino-cross sheep (2–5 years old, body weight 95 ± 12 kg) without orthopedic disease euthanized for reasons unrelated to this study, were cultured in complete StemMACS medium supplemented with MSC Expansion media supplement XF and 1% Pen/Strep under standard conditions (37 °C, 20% O_2_, 5% CO_2_, humidified incubator)^34^. The sheep from which the cells were obtained had been euthanized with approval by the institutional ethics and animal welfare committee and the national authority (ethical approval number 68.205/0100-V/3b/2018), in compliance with all relevant guidelines and regulations including the ARRIVE guidelines. Euthanasia had been conducted following sedation with detomidine and butorphanol, placement of a catheter in the jugular vein, and induction of general anesthesia with thiopental, by the administration of T61, a veterinary euthanasia drug containing tetracaine hydrochloride, mebezonium iodide, and embutramide.

All assays were performed with three biological replicates per group, using primary chondrocytes in passages 2–3. For qPCR and proliferation assays, 1000 cells per well were seeded on 96-well plates with six technical replicates per donor, which were pooled for qPCR. For wound healing (scratch assays) and cell viability (cell metabolic activity, MTT assay), 5000 cells were seeded per well on gelatine-coated 96-well plates with three technical replicates per donor (see Supplementary Material for detailed methodology). For 3D culture, 300,000 chondrocytes were pelleted in 15mL falcon tubes and incubated undisturbed for 72 h before subsequent experiments.

### Comparison of dexamethasone versus triamcinolone treatment

The effects of varying doses of DEX (1nM, 10nM, 40nM^28,31,32^, 1µM, Sigma Aldrich, Germany) and TA (38µM, 76µM, 152µM, Dermapharm, Austria)^35,36^ on inflamed chondrocytes were assessed using viability and proliferation assays and expression of inflammatory and ECM-related genes as read-outs. After chondrocytes in 2D culture were inflamed with 1ng/mL of interleukin-1 β (IL-1β) and 1ng/mL of tumor necrosis factor-α (TNF-α) (ImmunoTools, Germany) for 24 h, they received fresh medium containing these inflammatory cytokines along with corticosteroid treatment (T0). Healthy, non-inflamed and inflamed non-treated chondrocytes served as controls. The effect of DEX and TA on chondrocyte viability, proliferation and wound healing capacity was assessed for 48 h (T48). In addition, cells were harvested for gene expression analysis 24 h after treatment (T24).

### Treatment effect of dexamethasone (40nM) on chondrocytes

The effect of DEX at a therapeutic (40nM) concentration on the viability, proliferation and would healing capacity of healthy and inflamed chondrocytes was evaluated over 48 h. Additionally, its impact on the expression of inflammatory and ECM-related genes and senescence-associated β-galactosidase (SA-β-Gal) activity was assessed in 2D-cultured cells at 24 and 48 h (T24 and T48). SA-β-Gal activity was measured using a cellular senescence assay kit (Fluorometric format, Cell Biolabs, USA) according to the manufacturer’s protocol and normalized to total protein concentration measured using the Qubit Protein Assay Kit and Qubit 4 Fluorometer (Thermo Fisher Scientific, USA).

Healthy chondrocytes (HC), inflamed untreated (IC), and inflamed cells treated with an established senescence-inducing high-dose of DEX (1µM, hDIC) served as controls.

### In-depth analysis of the therapeutic effect of Dexamethasone (40nM) on inflamed chondrocytes - Next-generation sequencing and High-resolution mass spectrometry

HC, IC and inflamed DEX (40nM) treated chondrocytes (tDIC) in 3D pellet culture were harvested at T24 and T48 for high-resolution mass spectrometry proteomics and next-generation sequencing (NGS) analysis (see Suppl. Materials for detailed methodology)^37,38^.

Differentially expressed genes (DEGs) were further analyzed using Ingenuity Pathway Analysis (IPA, Qiagen, USA) to identify enriched pathways, upstream regulators, and potential network connections associated with the identified DEGs.

### Effects of repeated dexamethasone (40nM and 1µM) treatment

To assess the safety of repeated administrations of DEX, healthy and inflamed chondrocytes were treated three times every 72 h over a 9-day period, either with a therapeutic dose (40nM) or an established senescence-inducing high-dose of DEX (1µM)^29,32^. HC served as control. Due to the length of the assay, only 150 cells per well were seeded in 96-well plates. Proliferation was monitored throughout the 9-day culture period with DEX. Cell viability, senescence-associated β-galactosidase (SA-β-Gal) and caspase 3/7 activity, intracellular reactive oxygen species (ROS) levels and expression of senescence-associated genes were assessed 72 h post-third DEX application.

Intracellular ROS levels were analyzed using the OxiSelect™ Intracellular ROS assay Kit (Green Fluorescence; Cell Biolabs, USA) following the manufacturer’s instructions. ROS concentration was determined by comparison with a standard curve of 2’, 7’- Dichlorodihydrofluorescein (DCF) in cell culture medium without fetal calf serum (FCS).

Caspase-3/7 activity, a marker of apoptosis, was assessed using the Caspase-Glo^®^ 3/7 Assay System (Promega, USA) according to the manufacturer’s instructions and normalized to total protein concentrations.

### Statistical analysis

Data, excluding NGS and proteomics data, were analyzed using GraphPad Prism (version 8.4.3). Proliferation and wound healing data were analyzed using two-way ANOVA, all other experiments (qPCR, MTT, SA-β-Gal activity, ROS, Caspase 3/7) by one-way ANOVA with paired analysis. Statistical significance was defined as *p*-value < 0.05.

## Results

### Comparison of dexamethasone versus triamcinolone treatment

DEX at a concentration of 40nM was the only treatment that led to a significant downregulation of IL-6 and matrix metalloproteinase-3 (MMP-3) compared to IC. DEX at lower (1 and 10nM) and higher (1µM) dosages and TA in all doses (38, 76 and 152µM) failed to ameliorate the effect of the cytokines on inflammatory gene expression (Suppl. Figure 1). Neither DEX nor TA showed a significant impact on the viability (Suppl. Figure 2 C and D) or proliferation of inflamed cells (Suppl. Figure 3 A and B). Overall proliferation rates were highest in HC and lowest in IC (Suppl. Figure 3 A and B).

Based on these results, DEX at a concentration of 40nM was identified as a treatment that elicited a reliable anti-inflammatory response without evident deleterious effects.

### Treatment effect of dexamethasone (40nM) on chondrocytes

DEX treatment at a therapeutic (40nM) or high (1µM) dose had no significant influence on cell viability (Suppl. Figure 2 A and C), proliferation (Suppl. Figure 3 A) or wound healing (Suppl. Figure 4 A) in either healthy or inflamed chondrocytes. As expected^39^, SA-β-Gal activity increased with culture duration and confluence between 24 h and 48 h (Suppl. Figure 4B). However, a single treatment with DEX did not affect SA-β-Gal activity in HC or IC at either concentration (40nM or 1 µM) or time point (Suppl. Figure 4B). DEX treatment (40nM) significantly reduced the expression of inflammatory mediators IL-6 and MMP-3 in inflamed chondrocytes at T24. MMP-1 expression was non-significantly downregulated (Suppl. Figure 4 C). Interestingly, DEX treatment (40nM) of healthy chondrocytes (tDHC) also significantly decreased MMP-1 at T48 compared to HC. Additionally, DEX treatment (40nM) of healthy chondrocytes significantly downregulated aggrecan (ACAN) and non-significantly collagen type 2 (COL2) expression compared to HC (Suppl. Figure 4 C).

### In-depth analysis of the therapeutic effect of Dexamethasone (40nM) on inflamed chondrocytes - Next-generation sequencing and High-resolution mass spectrometry

Inflammatory stimulation resulted in differential expression of 56 DEGs (43 up- and 13.

downregulated) compared to the HC (“IC-HC”) at T24 (Table 1, Suppl. Figure 5) and 111 DEGs (56 up- and 55 down-regulated) at T48 (Suppl. Table 1, Suppl. Figure 5).

**Table 1:** Top 5 up- and down-regulated differentially expressed genes (DEGs) in inflamed untreated (IC) vs. healthy (HC) chondrocytes, inflamed DEX (40nM) treated chondrocytes (tDIC) vs. HC and tDIC vs. IC at 24 h (sorted by ascending LogFC value).

Gene ID	Gene name	logFC	logCPM	F	*P* Value	FDR
Top 5 Up DEGs in the IC vs. HC 24 h						
ENSOARG00020002903	CXCL6	10.31	6.91	460.61	5.82E−11	3.39E−07
ENSOARG00020002961	CXCL2	9.81	7.49	153.37	5.95E−06	2.67E−03
ENSOARG00020002886	CXCL8	8.11	7.42	181.82	8.60E−−07	6.27E−04
ENSOARG00020019763	MMP12	5.26	5.83	95.86	1.11E−05	4.27E−03
ENSOARG00020019819	MMP1	4.53	7.98	138.37	8.31E−07	6.27E−04
Top 5 down DEGs in the IC vs. HC 24 h						
ENSOARG00020002410	COL2A1	−2.46	8.52	60.01	1.17E−05	4.27E−03
ENSOARG00020022697	CCNA2	−1.89	5.02	27.46	2.06E−04	3.03E−02
ENSOARG00020009294	NNAT	−1.74	7.13	48.12	1.54E−05	5.27E−03
ENSOARG00020026168	SMOC2	−1.71	6.01	43.58	2.50E−05	7.28E−03
ENSOARG00020025095	CAPN6	−1.65	5.82	29.85	1.43E−04	2.32E−02
Top 5 up DEGs in the tDIC vs. IC 24 h						
ENSOARG00020011839	CD24	6.25	4.71	106.46	2.09E−12	1.90E−09
ENSOARG00020006060	OXT	4.19	4.74	114.10	1.40E−26	3.18E−23
ENSOARG00020021170	PDK4	3.40	4.11	54.96	1.40E−13	2.11E−10
ENSOARG00020000163	RARRES1	2.84	2.71	14.03	1.80E−04	1.58E−02
ENSOARG00020022068	HSD11B2	2.66	3.31	18.43	1.77E−05	2.26E−03
Top 5 down DEGs in the tDIC vs. IC 24 h						
ENSOARG00020029769	MMP13	−3.65	6.32	236.59	5.18E−53	4.69E−49
ENSOARG00020009939	SYT12	−2.89	2.75	15.01	1.07E−04	1.05E−02
ENSOARG00020002942	CXCL1	−2.75	4.41	31.60	2.06E−08	7.19E−06
ENSOARG00020013770	LFNG	−2.13	3.05	11.52	6.90E−04	4.11E−02
ENSOARG00020002903	CXCL6	−2.13	7.16	31.74	2.10E−04	1.79E−02
Top 5 Up DEGs in the tDIC vs. HC 24 h						
ENSOARG00020002903	CXCL6	8.11	4.74	163.37	1.14E−09	7.30E−07
ENSOARG00020002961	CXCL2	7.66	5.06	205.59	2.16E−10	2.50E−07
ENSOARG00020011839	CD24	6.70	4.76	128.29	6.29E−09	3.30E−06
ENSOARG00020007334	CCL20	5.66	5.29	186.63	4.37E−10	3.60E−07
ENSOARG00020002886	CXCL8	5.31	4.89	28.00	2.32E−03	2.89E−02
Top 5 Down DEGs in the tDIC vs. HC 24 h						
ENSOARG00020025232	MKI67	−3.43	5.11	62.68	1.83E−06	2.57E−04
ENSOARG00020002410	COL2A1	−3.08	8.42	208.92	1.93E−10	2.50E−07
ENSOARG00020031973	PNPLA3	−2.80	4.23	41.83	8.82E−06	7.18E−04
ENSOARG00020003927	CCNB1	−2.60	5.02	28.26	2.59E−04	7.22E−03
ENSOARG00020021805	GALNT5	−2.59	4.76	46.50	4.74E−06	4.64E−04

A significance cut-off of false discovery rate (FDR)-corrected *p*-value < 0.05 and a fold change (FC) threshold of |FC| ≥ 1.2 were applied to filter the DEGs.

DEX treatment of inflamed chondrocytes resulted in 168 DEGs (69 up- and 99 downregulated) compared to untreated inflamed cells (“tDIC-IC”) at T24 (Table 1, Suppl. Figure 5) and 5 DEGs (4 up- and 1 downregulated) at T48 (Suppl. Table 1, Suppl. Figure 5), indicating a rapidly decreasing effect of DEX on ongoing inflammation. In contrast, the difference between DEX treated and healthy cells (“tDIC-HC”) increased over time from 666 DEGs (258 up- and 408 downregulated) at T24 (Table 1, Suppl. Figure 5) to 1317 DEGs (517 up- and 800 downregulated) at T48 (Suppl. Table 1, Suppl. Figure 5).

**Fig. 1:**
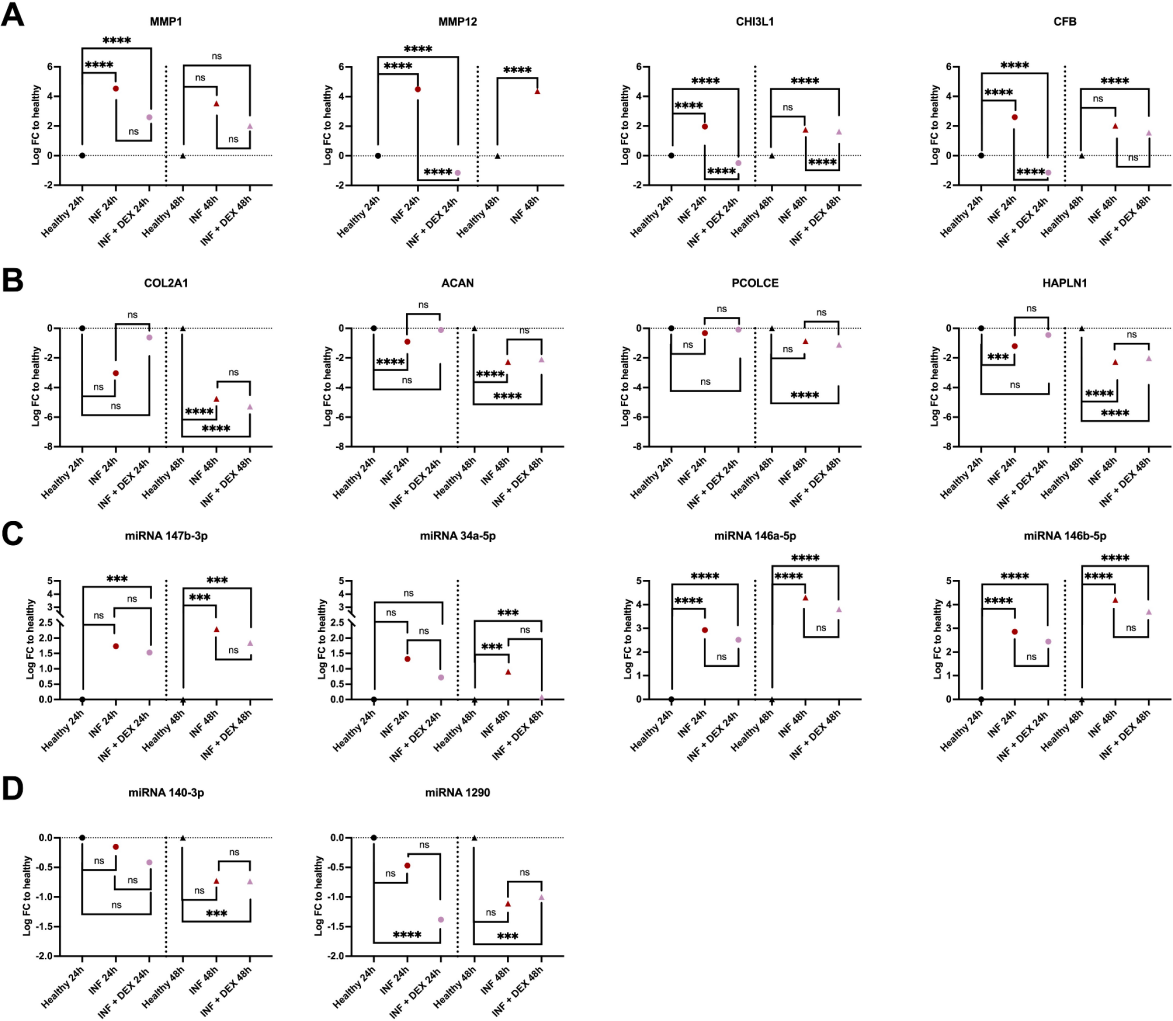
Treatment effect of Dexamethasone (DEX 40nM) on inflamed chondrocytes (mRNA, miRNA). (**A**) DEX 40nM treatment significantly reduced inflammation related genes like matrix metalloproteinase-1 (MMP-1: logFC=-1.91, FDR = 5.50E−01), matrix metalloproteinase-12 (MMP-12: logFC=−1.15, FDR = 8.80E−05), chitinase-3 like-protein-1 (CHI3L1: logFC=−0.50, FDR = 1.68E−03) and complement factor B (CFB: logFC=−1.15, FDR = 2.97E−04)). (**B**) Extracellular matrix (ECM)-related genes COL2A1 and ACAN showed similar significantly decreased expression in both DEX-treated and untreated inflamed chondrocytes compared to healthy controls at T48 (COL2A1: DEX treated: logFC=−5.28, FDR = 9.78E−08, untreated: logFC=−4.76, FDR = 3.56E−03; ACAN: DEX treated: logFC=−2.10 FDR = 1.53E−05, untreated: logFC=−2.28, FDR = 6.10E−03). (**C**) DEX treatment downregulated inflammation- related miRNAs miR-147-3p (T24: logFC=−0.13, FDR = 9.99E−01; T48: logFC=−0.29,FDR = 9.52E−01), miR-146a-5p (T24:logFC = 0.35,FDR = 9.99E−01; T48: logFC=−0.36, FDR = 9.52E−01), miR-146b-5p (T24: logFC=−0.35, FDR = 9.99E−01; T48: logFC=−0.36 ,FDR = 9.52E−01) and miR-34a-5p (T24: logFC = 0.08, FDR = 9.99E−01; T48: logFC=−0.08, FDR = 9.52E−015) compared to inflamed untreated controls. (**D**) DEX treatment significantly downregulated pro-regenerative miRNAs miR-140-3p (logFC=−0.73, FDR = 2.30E−02) and miR-1290 (logFC=−1, FDR = 2.40E−02) in DEX-treated cells compared to healthy chondrocytes at T48.

DEX 40nM treatment significantly reduced the inflammatory response in inflamed chondrocytes at T24 compared to untreated controls. This was indicated by the downregulation of genes associated with inflammation, including MMP-1, MMP-12, chitinase-3 like-protein-1 (CHI3L1) and complement Factor B (Fig. 1A; Table 1). By T48, the anti-inflammatory effect of DEX had mostly subsided, with only 5 DEGs remaining between DEX-treated and untreated inflamed chondrocytes (Suppl. Table 1). DEX treatment at 40nM also restored the expression of ECM-related factors, including Col2A1 and ACAN, which had been downregulated by inflammation, bringing them closer to healthy control levels at T24 (Fig. 1B; Table 1). However, this effect was transient, with the expression of these ECM-related genes in both DEX-treated and inflamed untreated chondrocytes returning to similarly downregulated levels compared to healthy cells by T48 (Fig. 1B, Suppl. Table 1).

MiRNA data mirrored the mRNA findings. DEX treatment downregulated inflammation-related miRNAs miR-147-3p, miR-146a-5p, miR-146b-5p and miR-34a-5p compared to inflamed untreated controls (Fig. 1C; Table 2). However, the degree of downregulation decreased between T24 and T48. In contrast to the effects on inflammatory miRNAs, DEX treatment significantly downregulated pro-regenerative miRNAs miR-140-3p and miR-1290 in T48 DEX-treated cells compared to healthy chondrocytes (Fig. 1D; Table 2).

**Table 2:** Top 5 differentially expressed (DE) MiRNA in inflamed untreated (IC) vs. healthy (HC) chondrocytes, inflamed DEX (40nM) treated chondrocytes (tDIC) vs. HC and tDIC vs. IC at 24 h and 48 h (sorted by ascending LogFC value).

miRNA	logFC	*P* Value	FDR
Top 5 DE miRNA in the IC vs. HC 24 h			
hsa-miR-146a-5p	2.93	2.20E−12	5.31E−10
hsa-miR-146b-5p	2.86 C	5.84E−12	7.03E−10
Top 5 DE miRNA in the tDIC vs. HC 24 h			
hsa-miR-146a-5p	2.52	1.71E−20	3.85E−18
hsa-miR-146b-5p	2.44	1.41E−19	1.59E−17
hsa-miR-147b-3p	1.53	4.65E−04	2.09E−02
hsa-miR-3195	-1.38	2.90E−04	1.63E−02
Top 5 DE miRNA in the IC vs. HC 48 h			
hsa-miR-146b-5p	4.2	8.50E−06	1.40E−03
hsa-miR-146a-5p	4.3	1.10E−05	1.40E−03
hsa-miR-147b-3p	2.3	5.30E−04	4.30E−02
hsa-miR-34a-5p	0.91	6.70E−04	4.30E−02
Top 5 DE miRNA in the tDIC vs. HC 48 h			
hsa-miR-146a-5p	3.8	3.30E−09	3.90E−07
hsa-miR-146b-5p	3.7	2.40E−09	3.90E−07
hsa-miR-147b-3p	1.8	1.20E−03	2.90E−02
hsa-miR-10400-5p	1.4	2.60E−03	4.10E−02
hsa-miR-34c-5p	1.3	1.20E−03	2.90E−02

A significance cut-off of false discovery rate (FDR)-corrected p-value < 0.05 and a fold change (FC) threshold of |FC| ≥ 1.2 were applied to filter the DE MiRNAs.

Venn analysis identified 10 DEGS shared by all three comparisons (IC-HC, tDIC-IC, tDIC-HC) at T24. The largest overlap (74 DEGs) was observed between “tDIC-IC " and “tDIC-HC”. A smaller overlap (36 DEGs) existed between “IC-HC” and “tDIC-HC”, indicating genes differentially expressed under both inflammatory and DEX-treated conditions compared to healthy controls. Only 3 DEGs were shared between “tDIC-IC” and “IC-HC”. Notably, “tDIC-IC” had a significantly higher number of unique DEGs (81) compared to “IC-HC” (7) and “tDIC-HC” had the most unique DEGs (546), indicating a greater effect of DEX treatment than inflammation on chondrocyte health (Suppl. Figure 5).

At T48, no DEGs were shared across all comparisons, and only a minimal overlap (3 DEGs) was observed between “tDIC-IC” and “tDIC-HC“(Suppl. Figure 5). While “tDIC-IC” and “IC-HC” shared no DEGs, 96 DEGs overlapped between “IC-HC” and “tDIC-HC”. Similar to T24, “IC-HC” had a small number of unique DEGs (15) and “tDIC-IC” had only 2. The most prominent difference was again in “tDIC-HC” with a substantial number of unique DEGs (1218), reinforcing the stronger and more persistent influence of DEX treatment on chondrocyte gene expression compared to inflammatory stimulation (Suppl. Figure 5).

**Fig. 2:**
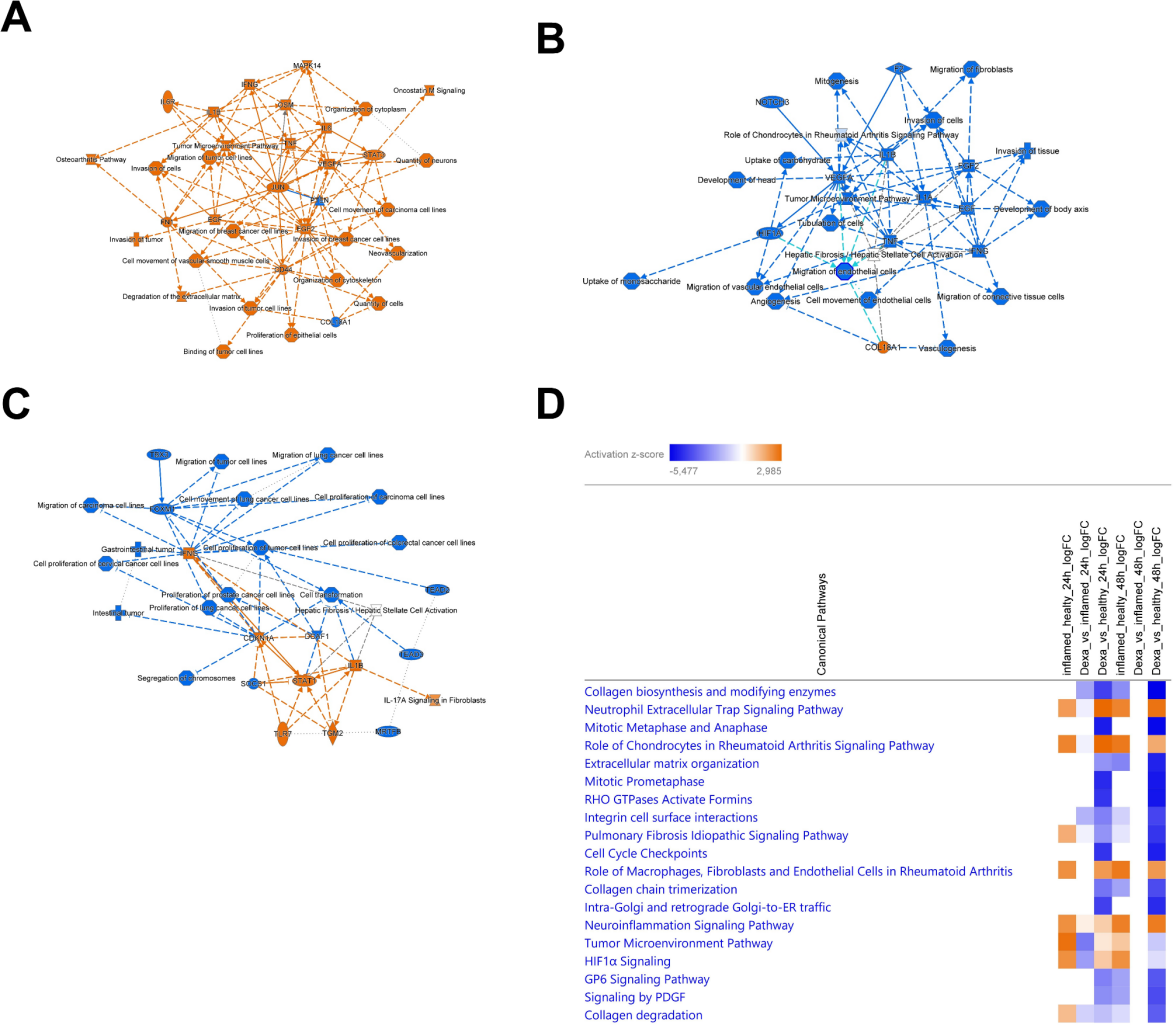
Ingenuity Pathway Analysis of differentially expressed genes at 24 h. (**A**) Graphical summary of of the Ingenuity Pathway Analysis (IPA) core analysis for genes differentially expressed between inflamed and healthy chondrocytes at T24. The graphical summary selects and connects a subset of the most significant entities predicted in the analysis, including canonical pathways, upstream regulators, diseases and biological functions, to visualize related biological activities. (**B**) Graphical summary of the genes differentially regulated between DEX treated and untreated inflamed chondrocytes at T24. (**C**) Graphical summary of the genes differentially regulated between DEX treated and healthy chondrocytes at T24. (**D**) Comparative analysis of the canonical pathways enriched in the DEGs of all 3 groups (healthy, inflamed untreated, DEX treated inflamed) at both time points (T24 and T48). Pathways are ranked accordingly to their z-score. orange … predicted activation (based on the z-score), blue … predicted inhibition, solid line: direct interaction, dashed line: indirect interaction;

Ingenuity Pathway Analysis (IPA) identified distinct significantly (*p* < 0.05, -log(p) > 1.3) enriched pathways in the DEGs between IC and HC at T24 (Fig. 2A, Suppl. Table 2) and T48 (Suppl. Figure 6 A, Suppl. Table 3), suggesting a dynamic time-dependent response in chondrocytes to inflammation.

Pathway analysis on DEGs between tDIC and IC at T24 (Fig. 2B, Suppl. Table 4) and T48 (Suppl. Table 5) suggest a transient therapeutic effect of DEX treatment. Initially, DEX exhibits anti-inflammatory effects, with significant enrichment and predicted inhibition of pathways such as “the role of chondrocytes in rheumatoid arthritis,” and the “osteoarthritis pathway.” However, by T48, only five genes are differentially expressed compared to IC, despite the persistence of the inflammatory response, raising concerns about DEX’s efficacy in completely resolving inflammation (Suppl. Table 1).

The genes differentially expressed in tDIC versus HC were significantly enriched in several pathways at both T24 (Fig. 2C, Suppl. Table 6) and T48 (Suppl. Figure 6B, Suppl. Table 7). Notably, pathways associated with cell cycle progression and mitosis were prominently enriched with predicted inhibition at both time points, suggesting disruptions in cell division processes or imbalanced cell cycle regulation. Additionally, persistent significant enrichment with predicted activation of inflammatory pathways such as the “Osteoarthritis Pathway” at T24 and T48 indicate that DEX treatment of inflamed chondrocytes does not achieve full control of inflammation compared to HC. Furthermore, significant enrichment with predicted inhibition of “Collagen biosynthesis and modifying enzymes” at both time points and “Extracellular matrix organization” and “Glycosaminoglycan metabolism” at T48 highlights potential alterations in the synthesis or modification of collagen and the cartilage ECM. Comparison between the pathways enriched at the two different time points, showed enrichment of largely the same pathways, however stronger enrichment and inhibition of pathways associated with cell cycle progression and anabolism at T48.

Comparison of the top 20 differentially enriched pathways between the three conditions (HC, IC, tDIC) at the 2 timepoints revealed tDIC versus HC to have the most enriched pathways associated with inhibited matrix synthesis and cell cycle progression, while IC versus HC unsurprisingly had most inflammation associated pathways (Fig. 2D, Suppl. Tables 2–8).

**Fig. 3:**
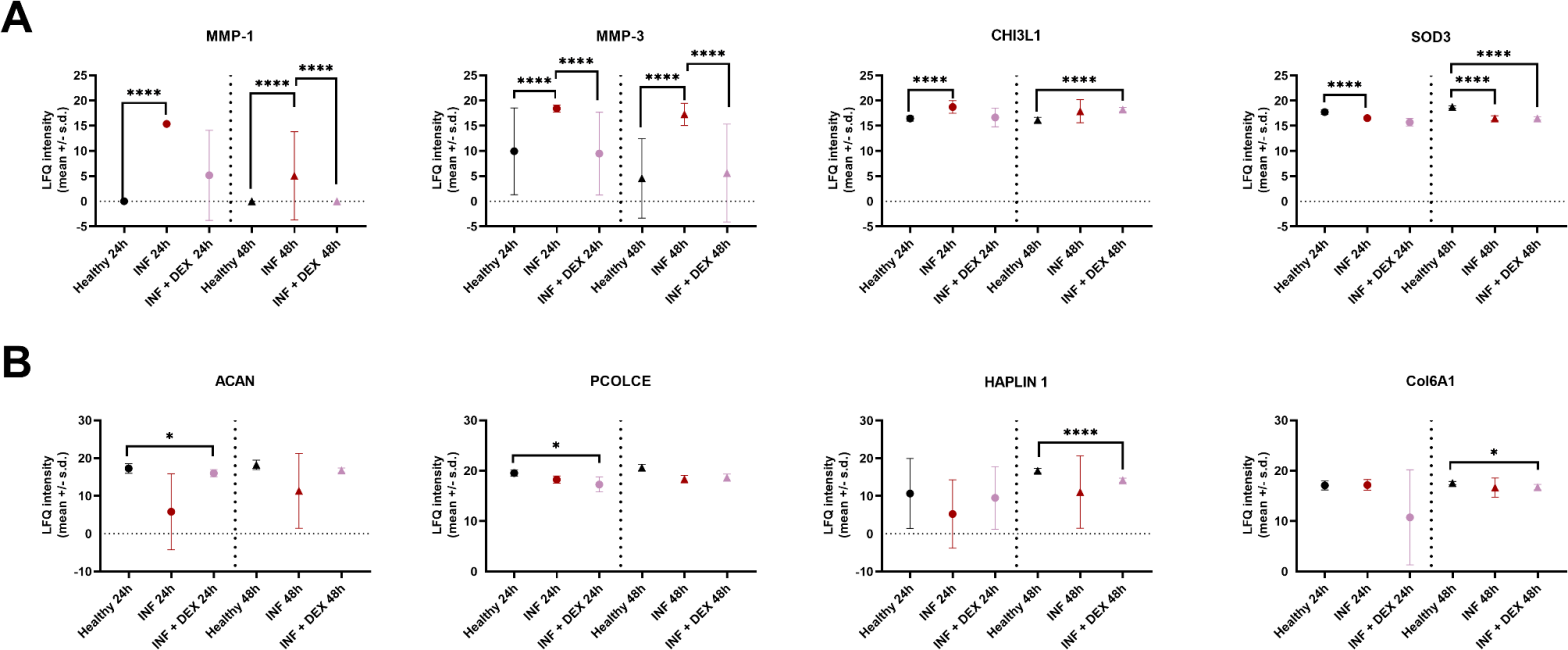
Treatment effect of Dexamethasone (DEX 40nM) on inflamed chondrocytes (proteomics data analysis). (**A**) DEX treatment led to a decreased abundance of inflammatory mediators such as matrix metalloproteinase − 1 (MMP1, T24: q = 0.746, T48: q = 0.000), matrix metalloproteinase − 3 (MMP3, T24: q = 0.000, T48: q = 0.000) and superoxide dismutase 3 (SOD3, T24: q = 0.072, T48: q = 1.00) compared to inflamed chondrocytes, but DEX treatment significantly increased chitinase-3 like-protein-1 (CH3L1, q = 0.000) at T48 compared to healthy cells. (**B**) DEX treatment non-significantly improved the secretion of extracellular matrix (ECM) proteins such as aggrecan (ACAN), procollagen C-proteinase enhancer (PCOLCE), hyaluronan and proteoglycan link protein 1 (HAPLIN1) and collagen type VI alpha 1 (COL6a1) compared to inflamed untreated chondrocytes, but their abundance remained significantly lower than in healthy controls at T24 (ACAN, T24: q = 0.048, T48: q = 0.283; PCOLCE, T24: q = 0.050, T48: q = 0.202) or T48 (HAPLIN1, T24: q = 0.349, T48: q = 0.000; COL6A1, T24: q = 0.276, T48: q = 0.048). ns…not significant (*p* ≥ 0.05), *…*p* < 0.05, **…*p* < 0.01, ***…*p* < 0.001.

High-resolution mass spectrometry identified a total of 395 proteins in the chondrocyte secretome (FDR ≤ 0.01). Among these, 128 proteins were confirmed to be genuinely secreted. DEX treatment led to a decreased abundance of inflammatory mediators such as MMP1, MMP3 and superoxide dismutase 3 (SOD3) compared to IC, although this decrease was not statistically significant, and the abundance of these proteins remained increased compared to HC (Fig. 3A). However, DEX treatment significantly increased CH3L1 expression at T48 compared to HC (Fig. 3A). In addition, the secretion of ECM proteins such as ACAN, procollagen C-proteinase enhancer, hyaluronan and proteoglycan link protein 1 and COL6A1 was numerically but non-significantly higher than in inflamed but lower compared to HC (Fig. 3B).

### Effects of repeated dexamethasone (40nM and 1µM) treatment

**Fig. 4:**
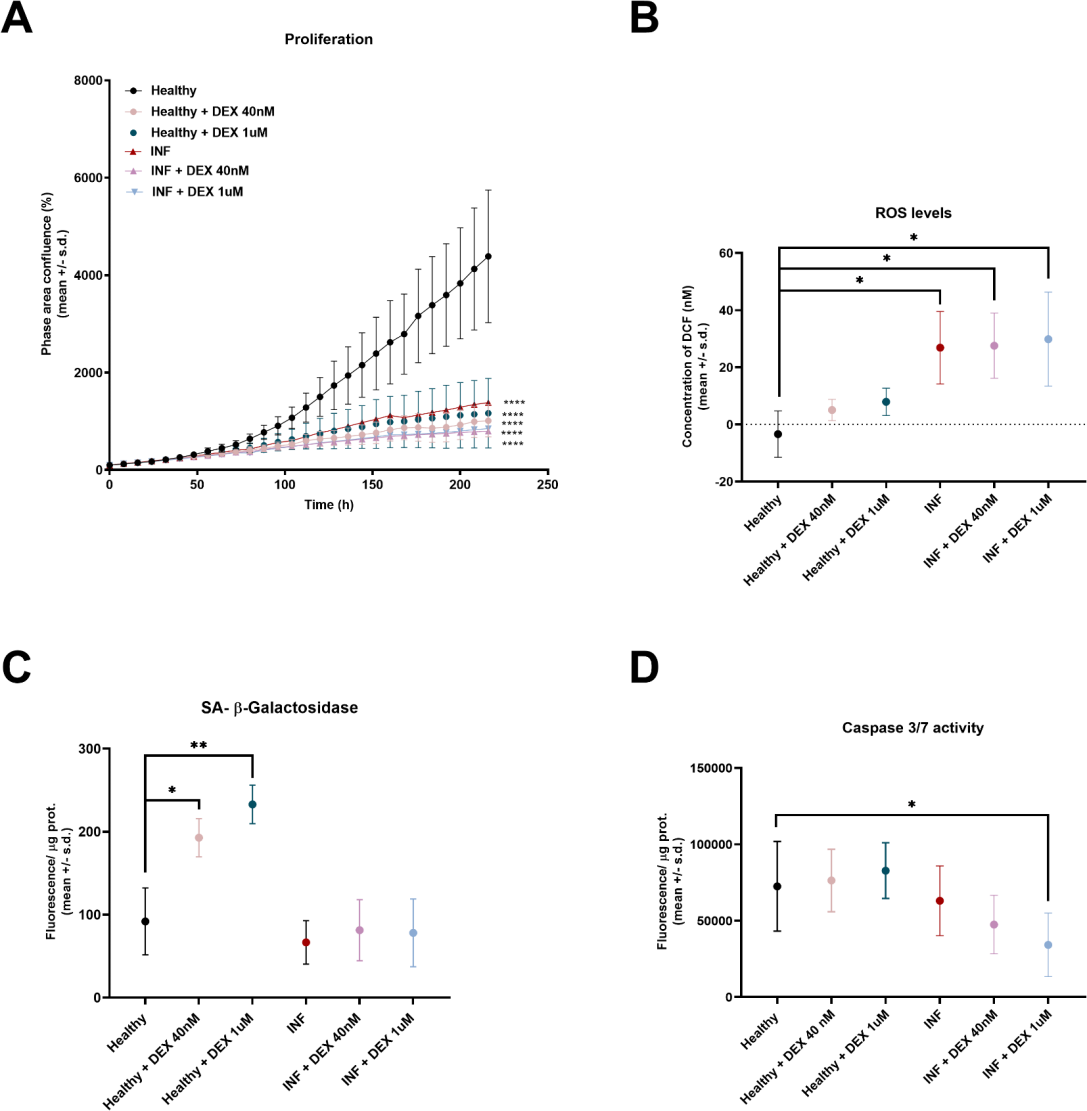
Long-term implications of Dexamethasone (DEX) (40nM and 1µM) treatments. (**A**) All conditions proliferated significantly slower (*p* < 0.0001) compared to healthy untreated over a period of 9 days. (**B**) Intracellular reactive oxygen species (ROS) levels significantly increased in inflamed (*p =* 0.0169) compared to healthy chondrocytes. DEX treatment did not reverse ROS accumulation in inflamed cells (40nM *p* = 0.8795, 1µM *p* = 0.4403), leaving ROS levels of DEX treated inflamed cells significantly higher than in healthy controls (40nM *p =* 0.0162, 1µM *p =* 0.0412). DEX treatment had no significant effect on ROS levels of healthy chondrocytes (40nM *p* = 0.1741, 1µM *p* = 0.0818). (**C**) SA-β Gal activity significantly increased in healthy chondrocytes treated with DEX (40nM:*p =* 0.0411 and 1µM:*p =* 0.0078) compared to healthy untreated chondrocytes. Neither inflammation (*p* = 0.3310) nor DEX treatment of inflamed chondrocytes (40nM:*p* = 0.8390, 1µM:*p* = 0.7526) increased SA-β Gal activity compared to healthy chondrocytes. (**D**) Caspase 3/7 activity significantly decreased in inflamed (*p* = 0.0401) but not in healthy (*p* = 0.5299) chondrocytes treated with DEX 1µM compared to healthy controls. Neither inflammation itself (*p* = 0.6766) nor DEX treatment 40nM significantly altered caspase 3/7 activity in either inflamed (*p* = 0.1282) or healthy (*p* = 0.9497) chondrocytes. ns…not significant (*p* ≥ 0.05), *…*p* < 0.05, **…*p* < 0.01, ***…*p* < 0.001.

After 9 days of culture, cell viability remained unaltered in all comparison groups compared to HC (Suppl. Figure 7 A). However, all conditions proliferated significantly slower compared to HC (Fig. 4A).

Inflammation significantly increased intracellular ROS compared to HC (*p =* 0.0169, Fig. 4B). DEX treatment could neither reverse ROS elevation in IC, leaving ROS levels elevated compared to HC nor significantly affect ROS levels in HC (Fig. 4B).

SA-β Gal activity significantly increased in HC following repeated DEX administration at both the therapeutic (tDHC) and established senescence-inducing dose (hDHC) compared to HC (Fig. 4C). In contrast, surprisingly, neither inflammatory stimulation nor repeated DEX treatment of inflamed chondrocytes increased SA-β Gal activity compared to HC (Fig. 4C). The stronger senescence-inducing effect of DEX administration on HC compared to IC was also confirmed by the expression of senescence-associated gene p53 (Suppl. Figure 7B). Treatment of healthy but not inflamed chondrocytes with 1µM DEX led to a significant upregulation of p53, but not p21 or sirt1 (Suppl. Figure 7B). In contrast, DEX 40nM treatment of inflamed but not healthy chondrocytes promoted a significant upregulation of sirt1 (*p* = 0.0308), but not p53 or p21 (Suppl. Figure 7B). Senescence-associated gene expression was not significantly affected by inflammation.

Caspase 3/7 activity significantly decreased in inflamed but not healthy chondrocytes treated with high-dose (1µM) DEX compared to HC (Fig. 4D). Neither inflammation itself nor DEX treatment at 40nM significantly altered caspase 3/7 activity in either inflamed or healthy chondrocytes (Fig. 4D).

## Discussion

Dexamethasone at a single dose of 40nM effectively but only transiently reduced inflammation. In contrast, TA, one of the GC most injected intraarticularly to treat OA, applied at the dose range of 10–40 mg/ml (= 25.5–102 µM) typically used for intraarticular therapy in clinics^10^, surprisingly did not ameliorate the effects of inflammation at any of the tested concentration (38, 76 and 152 µM) in this study. Clinically, typically 4 mg DEX, the equivalent to 9µM (based on an average knee synovial volume of 113 ml), are injected intraarticularly to treat OA^10^, which, given the difference in glucocorticoid potency, is equivalent to 20 mg triamcinolone^5,8,17^. Prior in vitro studies showed chondroprotective effects of DEX on inflamed chondrocytes at concentration ranges from 1nM to 100µM^4,5^. The 40nM DEX used as a therapeutic dose in this study is thus substantially lower than those used in typical clinical injections but in the dose range also proven effective against inflammatory stress in chondrocytes in other in vitro studies^4^. Yet, analogous to previous studies^8,19,40^, DEX yielded an imperfect rescue of inflamed chondrocytes, reducing pro-inflammatory mediators and catabolic factors but simultaneously exhibiting time-dependent detrimental effects on matrix production and collagen synthesis. Furthermore, the therapeutic effect of DEX on inflamed chondrocytes was very transient. While DEX displayed initial anti-inflammatory activity at T24, evidenced by 168 DEGs with significant enrichment and predicted inhibition of pathways like “Role of Chondrocytes in Rheumatoid Arthritis”, “Osteoarthritis Pathway” and “degradation of the extracellular matrix”, only 5 genes remained differentially expressed compared to untreated inflamed controls at T48 despite persistent inflammation. The transient anti-inflammatory effect observed in this study raises concerns about the efficacy of DEX for achieving complete and sustained inflammation resolution in chondrocytes. Studies assessing the anti-inflammatory effects of dexamethasone in vitro have predominantly evaluated the therapeutic effect at a single time point^4,7,10,12,19,20,23–27,31,40^. To our knowledge, to date no studies have compared the temporal effect of DEX on inflamed chondrocytes transcriptome and proteome at T24 and T48. Future investigations should explore the mechanisms underlying the transient effect identified in this study and identify potential strategies to improve DEX’s long-term therapeutic effectiveness or alternative therapeutic approaches for inflammatory joint diseases.

In contrast to the decreasing difference in gene expression between DEX treated and untreated inflamed chondrocytes, the difference between DEX-treated and healthy chondrocytes increased over time from 666 to 1317 DEGs. The fact that the difference between DEX-treated and healthy cells was far greater than between inflamed untreated and healthy cells (T24: 56, T48; 111 DEGs), suggests caution regarding the use of DEX to treat articular inflammation. This is further underscored by the distinct pathway enrichment patterns observed in these DEGs, which reveal a complex interplay between cell cycle regulation, inflammatory signaling, and ECM remodeling in DEX treated compared to healthy chondrocytes. Notably, pathways involved in all stages of the mitotic cell cycle and chromosomal replication were significantly enriched and predicted to be inhibited at both T24 and T48. The upregulation of cell death, cellular stress and catabolic pathways and downregulation of ECM synthesis pathways also suggest potential detrimental effects on cell proliferation, differentiation, and matrix homeostasis. Overall, the data indicates that DEX treatment causes chondrocytes to undergo stress and may lead to senescence and ECM degradation. Further investigation is warranted to elucidate the specific downstream effects of DEX on these pathways and their impact on chondrocyte function.

Previous studies on the effects of DEX on collagen synthesis have reported conflicting results^4,8,25–27,31^. Investigations on inflamed cartilage explants report large positive effects of DEX at doses ranging from 1nM to 100µM, with a decrease in glycosaminoglycan loss and gene expression of inflammatory and catabolic genes, supporting the hypothesis that DEX helps to prevent the degradation of ECM^4,8,20,27^. In inflamed chondrocytes, DEX treatment in the same dose range also exhibited largely beneficial effects, reducing the gene expression of matrix proteases, cyclooxygenase-2 (COX2) and prostaglandin E2, but also of tissue-inhibitors of metalloproteinases (TIMPS), indicating that DEX may have a dual effect on the regulation of matrix remodeling^7,8,23,27^.

In contrast to the largely beneficial effects of DEX under inflamed conditions, application of DEX to healthy cartilage explants and chondrocytes results in time- and dose-dependent detrimental effects on cartilage, ranging from decreased cell proliferation, metabolic activity, and ECM synthesis to increased autophagy, cell death or senescence^4,7,8,25–27,29,31,41^. In vivo data confirm the in vitro results with intraarticular DEX treatment protecting against glycosaminoglycan loss and ECM proteolysis when applied peritraumatically but leading to matrix degeneration and chondrocyte death in healthy tissue^6,8,42^. The treatment dose and duration and tissue-state dependent divergent effects of DEX highlight the importance of judicious use of DEX in OA therapy.

Repeated intra-articular GC injections are a common clinical practice for treating OA^6^. In fact, the FDA-approved dosage for intra-articular dexamethasone specifies 0.2-0.6 mg administered up to every three days (https://www.accessdata.fda.gov/drugsatfda_docs/label/2014/084916s066lbl.pdf, accessed May 2024). However, in this study detrimental effects of DEX on chondrocyte cell cycle progression and ECM homeostasis were observed already after a single treatment with the lowest DEX dose (40nM) that achieved an anti-inflammatory effect. Furthermore, previous studies demonstrated that long-term use of DEX exacerbates proteoglycan loss in articular cartilage and accelerates OA progression in vivo by enhancing extracellular matrix calcification and increasing chondrocyte senescence and apoptosis^28,29,43^.

DEX induces senescence through several interlinked mechanisms, each contributing to the complex process of cellular aging and growth arrest. DEX binds to glucocorticoid receptors, triggering a cascade of signaling events that modify the cell’s gene expression profiles and activate pathways associated with cell cycle arrest^30,44,45^. In addition, DEX elevates ROS levels through mitochondrial dysfunction, modulation of antioxidant defenses and various signaling pathways such as TGF-β/SMAD3-NOX4, leading to oxidative stress and DNA damage^30,44,45^. This damage activates the DNA damage response, particularly the p53 pathway, resulting in cell cycle arrest and the induction of senescence^30^.

Notably, repeated administration of DEX to healthy chondrocytes at concentrations of both 40nM and 1µM significantly increased SA-β Gal activity compared to untreated healthy cells. Interestingly, this effect was not observed in inflamed chondrocytes, although both untreated and DEX-treated inflamed chondrocytes exhibited significantly higher ROS levels compared to their healthy counterparts. As DEX induces senescence by increasing ROS levels via the TGFβ/SMAD3-NOX4 axis^44^, the lower senescence levels in inflamed DEX-treated cells may be due to inflammation inhibiting SMAD2/3 signaling in chondrocytes via SMAD linker (de)-phosphorylation^46^. Another explanation for the seemingly protective role of inflammation against senescence may lie in the inhibition of senescence and apoptosis through the activation of autophagy. Autophagy plays a dual and temporally variable role in OA pathogenesis^29,43,47^. While advanced OA is associated with decreased autophagy, both nutritional and catabolic (IL-1 stimulation) stresses transiently increase autophagy in chondrocytes as a protective mechanism to avoid cell death^29,31,47–50^. The inflamed chondrocytes in this study may thus have been protected from senescence by inflammatory stress-induced increased autophagy. Intriguingly, DEX has also been shown in previous studies to transiently increase autophagy in chondrocytes^31,43,47–49^. Chondrocytes exposed to up to 24 h treatment with 100µM DEX demonstrated an initial increase in autophagy, indicated by upregulation of LC-II, with a subsequent decrease of autophagy but corresponding rise in senescence already noted at 48 h after injury^31,49^. The rapid reduction of autophagy within 48 h after DEX treatment may contribute to the upregulation of senescence-associated DEGs and pathways at T48 compared to T24 in this study.

Neither long-term inflammation nor repeated DEX treatment significantly increased apoptosis marker Caspase 3/7. To the contrary, repeated administration of high-dose DEX to inflamed chondrocytes significantly decreased Caspase 3/7 levels compared to healthy controls, which supports findings from a previous study reporting decreased Caspase 3 activation in meningitic animals following DEX treatment^51^.

Further studies are needed to elucidate the precise mechanisms underlying DEX-induced senescence, quiescence or apoptosis in chondrocytes and to investigate potential sexual dimorphism in DEX effects due to androgen and glucocorticoid receptor homology and hormonal crosstalk.

## Conclusion

While DEX displayed a transient anti-inflammatory effect at a low dose, it also caused time-dependent detrimental effects on chondrocyte proliferation ECM homeostasis and promoted a senescence-like phenotype. These findings suggest caution regarding the use of DEX for treating OA, particularly considering the potential for long-term adverse effects.

## Electronic supplementary material

Below is the link to the electronic supplementary material.

Supplementary Material 1

Supplementary Material 2

Supplementary Material 3

Supplementary Material 4

Supplementary Material 5

Supplementary Material 6

Supplementary Material 7

Supplementary Material 8

Supplementary Material 9

Supplementary Material 10

## Data Availability

All data generated or analysed during this study are included in this published article and its supplementary information files or available from the corresponding author on reasonable request.

## Abbreviations

ACAN: Aggrecan
COL2: Collagen type 2
CHI3L1: Chitinase-3 like-protein-1
DEG: Differentially expressed genes
DEX: Dexamethasone
ECM: Extracellular matrix
GC: Glucocorticoids
HC: Healthy chondrocytes
hDHC: Healthy chondrocytes treated with a high-dose of DEX (1µM)
hDIC: inflamed chondrocytes treated with a high-dose of DEX (1µM)
IC: Inflamed untreated chondrocytes
IL-1β: Interleukin-1 β
MMP3: Matrix metalloproteinase-3
NGS: Next generation sequencing
OA: Osteoarthritis
ROS: Reactive oxygen species
SA-b-Gal: Senescence-associated β-galactosidase ()
T0/24/48: Time of treatment (T0), 24h/48/ after treatment
TA: Triamcinolone acetonide
tDIC: Inflamed chondrocytes treated with a therapeutic-dose of DEX (40nM)
tDHC: Healthy chondrocytes treated with a therapeutic-dose of DEX (40nM)
TNF-α: Tumor necrosis factor-α
