# Supplementary material for: Dexamethasone: a double-edged sword in the treatment of osteoarthritis
Source: Sci Rep. 2025 Apr 7;15:11832. doi: 10.1038/s41598-025-96050-2 (PMC11976973; doi:10.1038/s41598-025-96050-2)
Supplement: Supplementary file 1 — Supplementary Material 1 [file 41598_2025_96050_MOESM1_ESM.docx]

## Cell viability - MTT Assay

## Cell viability was assessed by adding 3-[4,5-dimethylthiazol-2-yl]-2,5 diphenyl tetrazolium bromide solution (MTT, Promega, USA) according to manufacturer’s instructions. The absorbance was measured with a plate reader (Varioskan LUX, Thermo Scientific, USA) at 595 nm.

## Cell Proliferation analysis

## The confluence of healthy and inflamed chondrocytes was determined using the IncuCyte® S3 Kinetic [Live Cell Imaging](https://www.sciencedirect.com/topics/medicine-and-dentistry/live-cell-imaging) System (Sartorius, Germany) which took pictures at a 2h interval for 48 h. Cell proliferation rates were calculated based on the increase of confluence levels using the IncuCyte® software (Sartorius, Germany).

## Wound healing assay

## The IncuCyte® WoundMaker tool (Sartorius, Germany) was used to create precise scratches (wounds) in the confluent chondrocyte monolayer. After inflicting the wounds, the cells were washed with PBS Mg2+/Ca2+ (Gibco Life technologies, UK) twice, followed by the addition of medium supplemented with 1ng/mL of interleukin-1 β (IL-1β) and 1ng/mL tumor necrosis factor-α (TNF-α). The chondrocyte monolayers were imaged every 2 h for a total of 48 h using the IncuCyte® live [cell imaging](https://www.sciencedirect.com/topics/biochemistry-genetics-and-molecular-biology/cellular-imaging) system (Sartorius, Germany) at 10X magnification. For each picture the wound width was calculated using the Scratch Wound analyses pipeline of the IncuCyte® software (Sartorius, Germany). Residual wound sizes were analysed for each timepoint, and wound closure rates were calculated.

## RNA extraction and Real Time Quantitative PCR

## For gene expression analysis adherent cells were lysed using a mixture of TRIzol reagent (Invitrogen, Thermo Scientific, Germany) + 1% β-Mercaptoethanol (Sigma-Aldrich, USA) (100µL/per well). Cells lysates from 2 x 3 wells were pooled per donor, snap frozen and stored at -80°C until further processing. After thawing, 200 µL of chloroform (Honeywell, US) were added to achieve phase separation (centrifugation 13,000g at 4°C for 15min) and RNA precipitation was performed by adding isopropyl alcohol (Sigma-Aldrich, USA) and glycerol (Thermo Scientific, Germany). The resulting mixture was incubated for 15 minutes on ice and then centrifuged for 15 minutes at 13,000g at 4°C. The obtained RNA pellet was washed twice with ice-cold 75% EtOH and dissolved in 20 µL of Nuclease-free water (Invitrogen, Thermo Scientific, Germany). Genomic DNA was removed using a DNA removal kit (Invitrogen, Thermo Scientific, Germany) for the removal of contaminating DNA following the user manual.

## For the qPCR reaction, RNA samples at a concentration of 1 ng/µL were used. The RevTrans QPCR One-Step EvaGreen kit (Bio&Sell, Feucht, Germany) was used for cDNA synthesis and the subsequent qPCR reaction according to the user manual. The reaction mixtures were incubated for 15 minutes at 50°C for cDNA generation, followed by the qPCR reaction: 95°C for 20 seconds, 59°C for 30 seconds and 72°C for 30 seconds. For each gene, a reaction mixture without the total RNA template was run as a negative control. The transcript data was analyzed using Agilent AriaMx 1.1 software (Agilent Technologies, Santa Clara, California, USA). The transcript level for the genes of interest was normalized to the transcript level of glyceraldehyde-3-phosphate dehydrogenase (GAPDH) and acidic ribosomal phosphoprotein P0 (RPLP0) and presented as a logarithmic fold change to the healthy condition. All primers were designed using Primer3 software. Specificity of the primers was analysed using the NCBI primer blast tool and in silico PCR tool of the UCSC genome browser.

## Library preparation and sequencing

## Gene expression analysis was performed as previously described (37). All primers were designed using Primer3 software. Specificity of the primers was analysed using the NCBI primer blast tool and in silico PCR tool of the UCSC genome browser.

## For next generation sequencing, total RNA was extracted using the miRNeasy Mini kit (Qiagen, Germany) and the QIAcube (Qiagen, Germany). RNA quality control was performed with the Bioanalyzer RNA 6000 kit (Agilent Technologies, US). Libraries for mRNA (QuantSeq 3’ mRNA-Seq V2 Library Prep Kit with UDI, Lexogen, Austria) small RNA (RealSeq Biofluids library preparation kit, RealSeq Biosciences, US) were prepared, pooled in equimolar ratio and sequenced on Illumina NovaSeq SP Flowcell (Illumina Innovative Technologies, USA) in SR100 mode. miND® Spike-Ins (TAmiRNA, Austria) were used in the small RNA library preparation for quality control and absolute quantification of miRNAs in the samples.

## For miRNA data, NGS data was analyzed using the miND® analysis pipeline and for mRNA analysis, adapter trimmed and quality filtered reads were aligned with STAR v2.7 against the genomic reference Oar-rambouillet.v2.0 provided by Ensembl (https://www.ensembl.org/Ovis_aries_rambouillet/Info/Index). Assignment of features to the mapped reads was performed with htseq-count v0.13 and differential expression (DE) analysis with edgeR v3.30. A significance cut-off of false discovery rate (FDR)-corrected p-value < 0.05 and a fold change (FC) threshold of |FC| ≥ 1.2 were applied to filter the differentially expressed genes (DEGs).

## Secretome profiling by high-resolution mass spectrometry

## High-resolution mass spectrometry was carried out as previously described (38). Data analysis including protein identification and label-free quantification (LFQ) was accomplished using MaxQuant software (version 1.6.17.0.22). Raw data were searched against the SwissProt database “homo sapiens” (version 141219, 20380 entries) including an allowed peptide tolerance of 20 ppm, a maximum of two missed cleavages, carbamidomethylation on cysteins as fixed modification as well as methionine oxidation and N-terminal protein acetylation as variable modification. A minimum of one unique peptide per protein was set as search criterium for positive identifications. The “match between runs” option was applied. For all peptide and protein identification a false discovery rate (FDR) ≤0.01 was set. Using Perseus software (version 1.6.14.0) identified proteins were filtered for reversed sequences and common contaminants. LFQ intensities were transformed (log2(x)), and proteins were additionally filtered for their number of independent identifications (protein identified in 70% of samples in at least one group).
