# Supplementary material for: Dexamethasone: a double-edged sword in the treatment of osteoarthritis
Source: Sci Rep. 2025 Apr 7;15:11832. doi: 10.1038/s41598-025-96050-2 (PMC11976973; doi:10.1038/s41598-025-96050-2)
Supplement: Supplementary file 2 — Supplementary Material 2 [file 41598_2025_96050_MOESM2_ESM.docx]

**Supplementary Tables**

Supplementary Table 1: Top 5 up- and down-regulated differentially expressed genes (DEGs) in inflamed untreated (IC) vs healthy (HC) chondrocytes, inflamed DEX (40nM) treated chondrocytes (tDIC) vs HC and tDIC vs IC at 48h (sorted by ascending LogFC value). A significance cut-off of false discovery rate (FDR)-corrected p-value < 0.05 and a fold change (FC) threshold of |FC| ≥ 1.2 were applied to filter the DEGs.

| **Top 5 Up DEGs in the INF vs H 48h** | | | | | | | |
| --- | --- | --- | --- | --- | --- | --- | --- |
| **Gene ID** | **Gene name** | **logFC** | **logCPM** | **F** | **PValue** | **FDR** |  |
| ENSOARG00020002903 | CXCL6 | 12.50 | 7.33 | 346.71 | 2.85E-10 | 2.04E-06 |  |
| ENSOARG00020032002 | CXCL3 | 8.10 | 6.67 | 70.61 | 3.52E-05 | 7.37E-03 |  |
| ENSOARG00020002961 | CXCL2 | 6.96 | 7.63 | 140.67 | 2.16E-08 | 5.14E-05 |  |
| ENSOARG00020007334 | CCL20 | 6.90 | 7.57 | 158.55 | 6.98E-09 | 2.50E-05 |  |
| ENSOARG00020019763 | MMP12 | 4.37 | 4.65 | 50.84 | 5.17E-06 | 2.05E-03 |  |
| **Top 5 Down DEGs in the INF vs H 48h** | | | | | | | |
| **Gene ID** | **Gene name** | **logFC** | **logCPM** | **F** | **PValue** | **FDR** |  |
| ENSOARG00020002410 | COL2A1 | -4.76 | 9.12 | 57.94 | 9.45E-06 | 3.56E-03 |  |
| ENSOARG00020027977 | SCRG1 | -4.04 | 5.76 | 100.60 | 8.47E-08 | 1.51E-04 |  |
| ENSOARG00020009184 | SYT8 | -4.01 | 3.54 | 24.41 | 2.19E-04 | 2.41E-02 |  |
| ENSOARG00020014096 | DES | -3.77 | 4.16 | 22.47 | 5.68E-04 | 4.36E-02 |  |
| ENSOARG00020024259 | MATN2 | -3.50 | 6.52 | 41.13 | 6.23E-05 | 1.04E-02 |  |
| **Top 5 Up DEGs in the INF DEX vs INF 48h** | | | | | | | |
| **Gene ID** | **Gene name** | **logFC** | **logCPM** | **F** | **PValue** | **FDR** |  |
| ENSOARG00020006060 | OXT | 4.83 | 4.75 | 63.32 | 4.01E-07 | 2.07E-03 |  |
| ENSOARG00020025716 | TRH | 4.26 | 4.01 | 35.48 | 1.38E-05 | 3.56E-02 |  |
| ENSOARG00020009778 | TGM2 | 3.14 | 8.15 | 66.03 | 2.39E-07 | 2.07E-03 |  |
| ENSOARG00020002697 | IL1R2 | 2.75 | 5.40 | 35.06 | 1.73E-05 | 3.57E-02 |  |
| **Top 5 Down DEGs in the INF DEX vs INF 48h** | | | | | | | |
| **Gene ID** | **Gene name** | **logFC** | **logCPM** | **F** | **PValue** | **FDR** |  |
| ENSOARG00020002942 | CXCL1 | -4.24 | 5.00 | 36.56 | 1.35E-05 | 3.56E-02 |  |
| **Top 5 Up DEGs in the INF DEX vs H 48h** | | | | | | | |
| **Gene ID** | **Gene name** | **logFC** | **logCPM** | **F** | **PValue** | **FDR** |  |
| ENSOARG00020002903 | CXCL6 | 10.44 | 5.31 | 235.47 | 6.92E-09 | 3.71E-06 |  |
| ENSOARG00020011839 | CD24 | 6.40 | 4.49 | 126.83 | 3.73E-08 | 1.20E-05 |  |
| ENSOARG00020032002 | CXCL3 | 6.26 | 4.53 | 68.77 | 1.58E-06 | 1.31E-04 |  |
| ENSOARG00020024251 | NOS2 | 5.65 | 7.88 | 427.35 | 1.85E-11 | 4.95E-08 |  |
| ENSOARG00020002961 | CXCL2 | 4.99 | 5.44 | 99.75 | 6.15E-07 | 6.97E-05 |  |
| **Top 5 Down DEGs in the INF DEX vs H 48h** | | | | | | | |
| **Gene ID** | **Gene name** | **logFC** | **logCPM** | **F** | **PValue** | **FDR** |  |
| ENSOARG00020013401 | ITGA8 | -8.02 | 3.12 | 38.89 | 5.82E-05 | 1.48E-03 |  |
| ENSOARG00020002410 | COL2A1 | -5.28 | 9.07 | 366.95 | 4.91E-11 | 9.87E-08 |  |
| ENSOARG00020017736 | PRSS35 | -5.05 | 7.04 | 469.22 | 1.01E-11 | 4.07E-08 |  |
| ENSOARG00020030934 | MSMP | -5.02 | 3.20 | 27.04 | 2.08E-04 | 3.72E-03 |  |
| ENSOARG00020018796 | IQGAP3 | -4.71 | 3.82 | 47.69 | 9.79E-06 | 4.17E-04 |  |

Supplementary Table 2: Top 20 Ingenuity Canonical Pathways enriched in the genes differentially expressed between inflamed and healthy chondrocytes at 24H (sorted by ascending absolute z-score)

| **Top 20 INF vs H 24H Ingenuity Canonical Pathways** | | | | |
| --- | --- | --- | --- | --- |
| **Ingenuity Canonical Pathways** | **-log(p-value)** | **Ratio** | **z-score** | **Molecules** |
| Role of Chondrocytes in Rheumatoid Arthritis Signaling Pathway | 6,72E00 | 3,55E-02 | 2,236 | CCL2,CXCL8,MMP1,MMP12,MMP3 |
| Tumor Microenvironment Pathway | 6,21E00 | 2,79E-02 | 2,236 | CCL2,CXCL8,MMP1,MMP12,MMP3 |
| Oncostatin M Signaling | 9,35E00 | 1,16E-01 | 2,000 | CHI3L1,EPAS1,MMP1,MMP3,TIMP3 |
| Role of Osteoblasts in Rheumatoid Arthritis Signaling Pathway | 4,12E00 | 1,64E-02 | 2,000 | CXCL8,MMP1,MMP12,MMP3 |
| Neuroinflammation Signaling Pathway | 3,68E00 | 1,26E-02 | 2,000 | CCL2,CXCL8,MMP3,SOD2 |
| Role of Macrophages, Fibroblasts and Endothelial Cells in Rheumatoid Arthritis | 3,6E00 | 1,2E-02 | 2,000 | CCL2,CXCL8,MMP1,MMP3 |
| Estrogen Receptor Signaling | 3,26E00 | 9,78E-03 | 2,000 | MMP1,MMP12,MMP3,SOD2 |
| S100 Family Signaling Pathway | 2,26E00 | 5,18E-03 | 2,000 | CXCL8,MMP1,MMP12,MMP3 |
| Osteoarthritis Pathway | 8,79E00 | 2,97E-02 | 1,890 | COL2A1,CXCL8,EPAS1,MMP1,MMP12,MMP3,TIMP3 |
| Pathogen Induced Cytokine Storm Signaling Pathway | 6 | 1,62E-02 | 1,633 | CCL2,COL2A1,CXCL6,CXCL8,FTH1,SRGN |
| Atherosclerosis Signaling | 6,85E00 | 3,76E-02 | 1,342 | CCL2,COL2A1,CXCL8,MMP1,MMP3 |
| Inhibition of Matrix Metalloproteases | 7,31E00 | 1,03E-01 | -1,000 | MMP1,MMP12,MMP3,TIMP3 |
| Collagen degradation | 6,51E00 | 6,56E-02 | 1,000 | COL2A1,MMP1,MMP12,MMP3 |
| Leukocyte Extravasation Signaling | 4,52E00 | 2,07E-02 | 1,000 | MMP1,MMP12,MMP3,TIMP3 |
| Role of Osteoclasts in Rheumatoid Arthritis Signaling Pathway | 3,73E00 | 1,3E-02 | 1,000 | COL2A1,MMP1,MMP12,MMP3 |
| Pulmonary Fibrosis Idiopathic Signaling Pathway | 3,64E00 | 1,23E-02 | 1,000 | COL2A1,MMP1,MMP12,MMP3 |
| IL-4 Signaling | 2,71E00 | 6,93E-03 | 1,000 | COL2A1,MMP12,TGM2,TIMP3 |
| Hematoma Resolution Signaling Pathway | 6,93E00 | 2,33E-02 | -0.816 | CXCL8,FTH1,MMP1,MMP12,MMP3,SOD2 |
| RAR Activation | 4,36E00 | 1,17E-02 | -0.447 | CXCL8,MMP1,MMP3,PDE4B,TGM2 |
| Hepatic Fibrosis Signaling Pathway | 4,42E00 | 1,2E-02 | 0.447 | CCL2,CXCL8,FTH1,MMP1,SOD2 |

Supplementary Table 3: Top 20 Ingenuity Canonical Pathways enriched in the genes differentially expressed between inflamed and healthy chondrocytes at 48H (sorted by ascending absolute z-score)

| **Top 20 INF vs H 48H Ingenuity Canonical Pathways** | | | | |
| --- | --- | --- | --- | --- |
| **Ingenuity Canonical Pathways** | **-log(p-value)** | **Ratio** | **z-score** | **Molecules** |
| Extracellular matrix organization | 6,93E00 | 6,6E-02 | -2,646 | ACAN,ASPN,COL11A1,COL2A1,COL9A1,NCAM1,TGFB3 |
| Role of Chondrocytes in Rheumatoid Arthritis Signaling Pathway | 6,08E00 | 4,96E-02 | 2,646 | ADAMTS4,CCL2,IL6,MMP12,MMP3,NFKB1,TLR2 |
| Role of Macrophages, Fibroblasts and Endothelial Cells in Rheumatoid Arthritis | 3,68E00 | 2,1E-02 | 2,646 | ADAMTS4,CCL2,IL6,MMP3,NFKB1,NFKBIA,TLR2 |
| Neuroinflammation Signaling Pathway | 6,64E00 | 3,15E-02 | 2,530 | BIRC3,CCL2,FAS,HMOX1,IL6,MMP3,NFKB1,SOD2,TGFB3,TLR2 |
| Neutrophil Extracellular Trap Signaling Pathway | 2,47E00 | 1,5E-02 | 2,449 | COL11A1,COL12A1,COL2A1,COL9A1,NFKB1,TLR2 |
| Osteoarthritis Pathway | 7,84E00 | 4,24E-02 | 2,333 | ACAN,ADAMTS4,COL2A1,EPAS1,ITGB8,MMP12,MMP3,NFKB1,TIMP3,TLR2 |
| Collagen biosynthesis and modifying enzymes | 5,35E00 | 7,46E-02 | -2,236 | COL11A1,COL12A1,COL2A1,COL9A1,P4HA3 |
| Colorectal Cancer Metastasis Signaling | 3,34E00 | 2,21E-02 | 2,236 | IL6,MMP12,MMP3,NFKB1,TGFB3,TLR2 |
| HIF1α Signaling | 3 | 2,39E-02 | 2,236 | CDKN1A,HMOX1,IL6,MMP12,MMP3 |
| Striated Muscle Contraction | 5,06E00 | 1,11E-01 | -2,000 | DES,TNNI2,TNNT3,TPM2 |
| Collagen chain trimerization | 4,71E00 | 9,09E-02 | -2,000 | COL11A1,COL12A1,COL2A1,COL9A1 |
| Signaling by PDGF | 4,23E00 | 6,9E-02 | -2,000 | COL2A1,COL9A1,THBS1,THBS2 |
| GP6 Signaling Pathway | 2,93E00 | 3,15E-02 | -2,000 | COL11A1,COL12A1,COL2A1,COL9A1 |
| Role of IL-17F in Allergic Inflammatory Airway Diseases | 4,59E00 | 8,51E-02 | 2,000 | CCL2,CXCL6,IL6,NFKB1 |
| Senescence-Associated Secretory Phenotype (SASP) | 3,84E00 | 5,48E-02 | 2,000 | CDKN1A,H2AC6,IL6,NFKB1 |
| TREM1 Signaling | 3,75E00 | 5,19E-02 | 2,000 | CCL2,IL6,NFKB1,TLR2 |
| Acute Phase Response Signaling | 3,24E00 | 2,7E-02 | 2,000 | HMOX1,IL6,NFKB1,NFKBIA,SOD2 |
| Coronavirus Pathogenesis Pathway | 2,19E00 | 1,96E-02 | 2,000 | CCL2,IL6,NFKB1,NFKBIA |
| IL-12 Signaling and Production in Macrophages | 2,02E00 | 1,75E-02 | 2,000 | NFKB1,TGFB3,THBS1,TLR2 |
| Neutrophil degranulation | 1,02E00 | 8,4E-03 | 2,000 | FTH1,NFKB1,PTX3,TLR2 |

Supplementary Table 4: Top 20 Ingenuity Canonical Pathways enriched in the genes differentially expressed between DEX treated and untreated inflamed chondrocytes at 24H (sorted by ascending absolute z-score)

| **Top 20 INF DEX vs INF 24H Ingenuity Canonical Pathways** | | | | |
| --- | --- | --- | --- | --- |
| **Ingenuity Canonical Pathways** | **-log(p-value)** | **Ratio** | **z-score** | **Molecules** |
| Triglyceride metabolism | 3,36E00 | 5,88E-02 |  | FABP4,GK |
| Role of IL-17F in Allergic Inflammatory Airway Diseases | 3,08E00 | 4,26E-02 |  | CXCL6,MMP13 |
| Role of IL-17A in Arthritis | 2,92E00 | 3,51E-02 |  | CXCL6,MMP13 |
| Docosahexaenoic Acid (DHA) Signaling | 2,84E00 | 1,2E-02 |  | FABP4,PLCD4,SYT12 |
| GPCR-Mediated Integration of Enteroendocrine Signaling Exemplified by an L Cell | 2,68E00 | 2,67E-02 |  | NMB,PLCD4 |
| Class A/1 (Rhodopsin-like receptors) | 2,55E00 | 9,46E-03 |  | CXCL6,NMB,TRH |
| Glycerol Degradation I | 2,27E00 | 1,67E-01 |  | GK |
| TR/RXR Activation | 2,23E00 | 1,56E-02 |  | SYT12,TRH |
| RAR Activation | 2,18E00 | 6,99E-03 |  | MMP13,PDE4C,TGM2 |
| Prednisone ADME | 2,09E00 | 1,11E-01 |  | HSD11B2 |
| Salvage Pathways of Pyrimidine Deoxyribonucleotides | 2,09E00 | 1,11E-01 |  | UPP1 |
| G alpha (q) signalling events | 1,97E00 | 1,15E-02 |  | NMB,TRH |
| Granulocyte Adhesion and Diapedesis | 1,9E00 | 1,06E-02 |  | CXCL6,MMP13 |
| Role of IL-17A in Psoriasis | 1,9E00 | 7,14E-02 |  | CXCL6 |
| PPARα/RXRα Activation | 1,88E00 | 1,03E-02 |  | GK,PLCD4 |
| Glucocorticoid Receptor Signaling | 1,82E00 | 5,15E-03 |  | FKBP5,MMP13,PDK4 |
| Agranulocyte Adhesion and Diapedesis | 1,81E00 | 9,52E-03 |  | CXCL6,MMP13 |
| Nucleotide salvage | 1,72E00 | 4,76E-02 |  | UPP1 |
| Digestion | 1,69E00 | 4,35E-02 |  | PIR |

Supplementary Table 5: Top 20 Ingenuity Canonical Pathways enriched in the genes differentially expressed between DEX treated and untreated inflamed chondrocytes at 48H (sorted by ascending absolute z-score)

| **Top 20 INF DEX vs INF at 48H Ingenuity Canonical Pathways** | | | | |
| --- | --- | --- | --- | --- |
| **Ingenuity Canonical Pathways** | **-log(p-value)** | **Ratio** | **z-score** | **Molecules** |
| Interleukin-10 signaling | 2,26E00 | 2,22E-02 |  | IL1R2 |
| PPAR Signaling | 1,88E00 | 9,35E-03 |  | IL1R2 |
| p38 MAPK Signaling | 1,83E00 | 8,33E-03 |  | IL1R2 |
| LXR/RXR Activation | 1,82E00 | 8,13E-03 |  | IL1R2 |
| IL-27 Signaling Pathway | 1,8E00 | 7,81E-03 |  | IL1R2 |
| TR/RXR Activation | 1,8E00 | 7,81E-03 |  | TRH |
| Interleukin-1 family signaling | 1,8E00 | 7,75E-03 |  | IL1R2 |
| IL-6 Signaling | 1,8E00 | 7,75E-03 |  | IL1R2 |
| STAT3 Pathway | 1,78E00 | 7,41E-03 |  | IL1R2 |
| Role of Chondrocytes in Rheumatoid Arthritis Signaling Pathway | 1,76E00 | 7,09E-03 |  | IL1R2 |
| IL-10 Signaling | 1,72E00 | 6,49E-03 |  | IL1R2 |
| Aryl Hydrocarbon Receptor Signaling | 1,71E00 | 6,29E-03 |  | TGM2 |
| G alpha (q) signalling events | 1,67E00 | 5,75E-03 |  | TRH |
| Granulocyte Adhesion and Diapedesis | 1,63E00 | 5,29E-03 |  | IL1R2 |
| Macrophage Alternative Activation Signaling Pathway | 1,63E00 | 5,26E-03 |  | IL1R2 |
| PPARα/RXRα Activation | 1,62E00 | 5,15E-03 |  | IL1R2 |
| Hepatic Fibrosis / Hepatic Stellate Cell Activation | 1,62E00 | 5,15E-03 |  | IL1R2 |
| PI3K/AKT Signaling | 1,61E00 | 0.005 |  | IL1R2 |
| ID1 Signaling Pathway | 1,61E00 | 4,98E-03 |  | TGM2 |
| Activin Inhibin Signaling Pathway | 1,59E00 | 4,74E-03 |  | IL1R2 |

Supplementary Table 6: Top 20 Ingenuity Canonical Pathways enriched in the genes differentially expressed between DEX treated inflamed and healthy chondrocytes at 24H (sorted by ascending absolute z-score)

| **Top 20 INF DEX vs H at 24H Ingenuity Canonical Pathways** | | | | |
| --- | --- | --- | --- | --- |
| **Ingenuity Canonical Pathways** | **-log(p-value)** | **Ratio** | **z-score** | **Molecules** |
| Mitotic Prometaphase | 5,57E00 | 3,94E-02 | -2828 | BIRC5,BUB1,CCNB1,CENPE,CENPH,NDC80,PLK1,PLK4 |
| Mitotic Metaphase and Anaphase | 5,1E00 | 3,4E-02 | -2828 | BIRC5,BUB1,CCNB1,CENPE,CENPH,NDC80,PLK1,UBE2C |
| RHO GTPases Activate Formins | 5,63E00 | 5,04E-02 | -2646 | BIRC5,BUB1,CENPE,CENPH,DIAPH3,NDC80,PLK1 |
| Chaperone Mediated Autophagy Signaling Pathway | 1,71E00 | 1,1E-02 | -2646 | BBC3,MDM2,MMP1,MMP12,MMP3,NFKBIA,PLIN2 |
| Role of Chondrocytes in Rheumatoid Arthritis Signaling Pathway | 5,59E00 | 4,96E-02 | 2646 | CXCL8,IL1R2,IL1RAP,MMP1,MMP12,MMP3,NOS2 |
| Role of Macrophages, Fibroblasts and Endothelial Cells in Rheumatoid Arthritis | 3,23E00 | 2,1E-02 | 2646 | CXCL8,IL1R2,IL1RAP,MMP1,MMP3,NFKBIA,NOS2 |
| Cell Cycle Checkpoints | 9,79E00 | 4,78E-02 | -2496 | BIRC5,BUB1,CCNA2,CCNB1,CDKN1A,CENPE,CENPH,MDM2,NDC80,NSD2,PLK1,RMI2,UBE2C |
| Kinetochore Metaphase Signaling Pathway | 6,32E00 | 6,36E-02 | -2449 | BIRC5,BUB1,CCNB1,CENPE,CENPH,NDC80,PLK1 |
| Hepatic Cholestasis | 3,39E00 | 2,69E-02 | 2449 | CXCL8,FOXO1,IL1R2,IL1RAP,NFKBIA,NOS2 |
| S100 Family Signaling Pathway | 9,42E-01 | 7,77E-03 | 2449 | CDKN1A,CXCL8,MMP1,MMP12,MMP3,NOS2 |
| Osteoarthritis Pathway | 6,09E00 | 3,81E-02 | 2333 | COL2A1,CXCL8,IL1R2,IL1RAP,MMP1,MMP12,MMP3,NOS2,TIMP3 |
| Neutrophil Extracellular Trap Signaling Pathway | 1,54E00 | 1,25E-02 | 2236 | COL2A1,COL8A2,COL9A1,CXCL8,NOS2 |
| Collagen biosynthesis and modifying enzymes | 3,7E00 | 5,97E-02 | -2000 | COL2A1,COL8A2,COL9A1,P3H2 |
| Regulation of mitotic cell cycle | 3,25E00 | 4,55E-02 | -2000 | CCNA2,CCNB1,PLK1,UBE2C |
| RHO GTPase cycle | 8,93E-01 | 8,89E-03 | -2000 | ARHGAP11A,DIAPH3,IQGAP3,WWP2 |
| Oncostatin M Signaling | 4,46E00 | 9,3E-02 | 2000 | CHI3L1,MMP1,MMP3,TIMP3 |
| IL-6 Signaling | 2,63E00 | 3,1E-02 | 2000 | CXCL8,IL1R2,IL1RAP,NFKBIA |
| PIP3 activates AKT signaling | 2,51E00 | 2,86E-02 | 2000 | CDKN1A,FOXO1,IL1RAP,MDM2 |
| Role of Osteoblasts in Rheumatoid Arthritis Signaling Pathway | 1,68E00 | 1,64E-02 | 2000 | CXCL8,MMP1,MMP12,MMP3 |
| Cachexia Signaling Pathway | 2,98E00 | 1,9E-02 | 1890 | CAPN6,CXCL8,FOXO1,GDF15,IL1R2,IL1RAP,NOS2 |

Supplementary Table 7: Top 20 Ingenuity Canonical Pathways enriched in the genes differentially expressed between DEX treated inflamed and healthy chondrocytes at 48H (sorted by ascending absolute z-score)

| **Top 20 INF DEX vs H at 48H Ingenuity Canonical Pathways** | | | | |
| --- | --- | --- | --- | --- |
| **Ingenuity Canonical Pathways** | **-log(p-value)** | **Ratio** | **z-score** | **Molecules** |
| Mitotic Prometaphase | 1,44E01 | 1,18E-01 | -4899.00 | BIRC5,BUB1,BUB1B,CCNB1,CCNB2,CDC20,CDK1,CENPA,CENPE,CENPF,CENPP,ERCC6L,HAUS7,INCENP,KIF2C,NCAPD2,NCAPG,NCAPH,NDC80,NUF2,PLK1,SGO1,TUBA1A,TUBB |
| Mitotic Metaphase and Anaphase | 1,03E01 | 8,94E-02 | -4583.00 | BIRC5,BUB1,BUB1B,CCNB1,CCNB2,CDC20,CDK1,CENPA,CENPE,CENPF,CENPP,ERCC6L,INCENP,KIF2C,NDC80,NUF2,PLK1,SGO1,TMPO,TUBA1A,UBE2C |
| RHO GTPases Activate Formins | 1,06E01 | 1,22E-01 | -4123.00 | BIRC5,BUB1,BUB1B,CDC20,CENPA,CENPE,CENPF,CENPP,DIAPH3,ERCC6L,INCENP,KIF2C,NDC80,NUF2,PLK1,SGO1,TUBA1A |
| Cell Cycle Checkpoints | 1,43E01 | 9,93E-02 | -4041.00 | BIRC5,BUB1,BUB1B,CCNA2,CCNB1,CCNB2,CDC20,CDC45,CDK1,CDKN1A,CENPA,CENPE,CENPF,CENPP,ERCC6L,INCENP,KIF2C,MCM2,MCM5,MDM2,MDM4,NDC80,NSD2,NUF2,PLK1,SGO1,UBE2C |
| Pulmonary Fibrosis Idiopathic Signaling Pathway | 7,07E00 | 6,13E-02 | -4025.00 | ACTA2,BBC3,BIRC5,COL11A1,COL12A1,COL16A1,COL1A1,COL1A2,COL2A1,COL5A2,COL8A2,COL9A1,FGFR2,FZD8,MAP2K6,MMP16,MMP23B,PDGFRA,PLAU,TGFB3 |
| Extracellular matrix organization | 1,04E01 | 1,42E-01 | -3873.00 | ACAN,ADAM12,ASPN,COL11A1,COL1A1,COL1A2,COL2A1,COL5A2,COL9A1,FMOD,ITGA1,ITGA8,NCAM1,TGFB3,TNN |
| Nuclear Cytoskeleton Signaling Pathway | 6,02E00 | 6,82E-02 | -3873.00 | ACTA2,CENPE,DES,ITGA1,ITGA8,KIF11,KIF15,KIF20A,KIF22,KIF23,KIF2C,KIF4A,KIFC1,RACGAP1,TUBA1A |
| Eicosanoid Signaling | 0 | 1,07E-02 | -3,679 | ABHD3,AP1G2,PLAAT3 |
| MSP-RON Signaling in Macrophages Pathway | 0 | 8,4E-03 | -3,678 | NOS2 |
| MSP-RON Signaling in Cancer Cells Pathway | 0 | 1,43E-02 | -3,677 | ACTA2,NFKBIA |
| Regulation of the Epithelial Mesenchymal Transition in Development Pathway | 0 | 1,15E-02 | -3,676 | FZD8 |
| Insulin Secretion Signaling Pathway | 0 | 3,64E-03 | -3,675 | PC |
| Xenobiotic Metabolism PXR Signaling Pathway | 0 | 5,13E-03 | -3,674 | NOS2 |
| Xenobiotic Metabolism General Signaling Pathway | 0 | 6,99E-03 | -3,673 | MAP2K6 |
| Inhibition of ARE-Mediated mRNA Degradation Pathway | 0 | 6,13E-03 | -3,672 | PPP2R2B |
| White Adipose Tissue Browning Pathway | 0 | 1,45E-02 | -3,671 | BMP7,FGFR2 |
| Systemic Lupus Erythematosus in B Cell Signaling Pathway | 0 | 4,15E-03 | -3,670 | CXCL8,NFATC4,TGFB3 |
| T Cell Exhaustion Signaling Pathway | 0 | 5,29E-03 | -3,669 | CD247,NFATC4,PPP2R2B |
| Apelin Endothelial Signaling Pathway | 0 | 1,42E-02 | -3,668 | APLN,CCL2 |
| Apelin Cardiomyocyte Signaling Pathway | 0 | 1,01E-02 | -3,667 | APLN |

Supplementary Table 8: Comparison of the top 40 canonical pathways identified by Ingenuity Pathway Analysis between the three comparison groups (healthy, inflamed untreated and DEX treated inflamed chondrocytes) at 48H (sorted by ascending absolute z-score of the DEX treated inflamed versus healthy chondrocyte comparison)

| **Top 40 Comparisons of Ingenuity Canonical Pathways between INF DEX, INF and H at 48H** | | | |
| --- | --- | --- | --- |
| **Canonical Pathways** | **Inflamed_healty_48h_logFC** | **Dex_vs_inflamed_48h_logFC** | **Dex_vs_healthy_48h_logFC** |
| Neutrophil Extracellular Trap Signaling Pathway | 2.449 | N/A | 2.795 |
| Sirtuin Signaling Pathway | N/A | N/A | 2.746 |
| Coronavirus Pathogenesis Pathway | 2 | N/A | 2.673 |
| iNOS Signaling | N/A | N/A | 2.646 |
| Neuroinflammation Signaling Pathway | 2.53 | N/A | 2.6 |
| Hepatic Cholestasis | 1 | N/A | 2.5 |
| IL-6 Signaling | N/A | N/A | 2.496 |
| Role of Pattern Recognition Receptors in Recognition of Bacteria and Viruses | N/A | N/A | 2.449 |
| NFE2L2 regulating anti-oxidant/detoxification enzymes | N/A | N/A | 2.449 |
| RHOGDI Signaling | N/A | N/A | 2.357 |
| NLR signaling pathways | N/A | N/A | 2.333 |
| Amino acids regulate mTORC1 | N/A | N/A | 2.333 |
| Acute Phase Response Signaling | 2 | N/A | 2.324 |
| WNT/β-catenin Signaling | N/A | N/A | 2.324 |
| FOXO-mediated transcription of cell death genes | N/A | N/A | 2.236 |
| ATF4 activates genes in response to endoplasmic reticulum stress | N/A | N/A | 2.236 |
| Autophagy | N/A | N/A | 2.236 |
| Role of MAPK Signaling in Promoting the Pathogenesis of Influenza | N/A | N/A | 2.138 |
| Induction of Apoptosis by HIV1 | 0.447 | N/A | 2.121 |
| MIF Regulation of Innate Immunity | N/A | N/A | 2.121 |
| Iron uptake and transport | N/A | N/A | 2.111 |
| Role of Macrophages, Fibroblasts and Endothelial Cells in Rheumatoid Arthritis | 2.646 | N/A | 2.058 |
| Response of EIF2AK1 (HRI) to heme deficiency | N/A | N/A | 2 |
| Regulation of TP53 Activity through Methylation | N/A | N/A | 2 |
| FOXO-mediated transcription of cell cycle genes | N/A | N/A | 2 |
| IL-17A Signaling in Gastric Cells | N/A | N/A | 2 |
| Interleukin-10 signaling | N/A | N/A | 2 |
| Role of Hypercytokinemia/hyperchemokinemia in the Pathogenesis of Influenza | N/A | N/A | 2 |
| 4-1BB Signaling in T Lymphocytes | N/A | N/A | 2 |
| Pexophagy | N/A | N/A | 2 |
| Production of Nitric Oxide and Reactive Oxygen Species in Macrophages | N/A | N/A | 1.941 |
| Dilated Cardiomyopathy Signaling Pathway | N/A | N/A | 1.941 |
| IL-33 Signaling Pathway | 1.342 | N/A | 1.897 |
| Role of IL-17F in Allergic Inflammatory Airway Diseases | 2 | N/A | 1.89 |
| TREM1 Signaling | 2 | N/A | 1.89 |
| IL-17A Signaling in Airway Cells | 1 | N/A | 1.89 |
| MAP kinase activation | N/A | N/A | 1.89 |
| Role of Chondrocytes in Rheumatoid Arthritis Signaling | 2.646 | N/A | 1.706 |
| IL-17 Signaling | 1.342 | N/A | 1.698 |
| DDX58/IFIH1-mediated induction of interferon-alpha/beta | N/A | N/A | 1.667 |
