# Supplementary material for: Dexamethasone: a double-edged sword in the treatment of osteoarthritis
Source: Sci Rep. 2025 Apr 7;15:11832. doi: 10.1038/s41598-025-96050-2 (PMC11976973; doi:10.1038/s41598-025-96050-2)
Supplement: Supplementary file 3 — Supplementary Material 3 [file 41598_2025_96050_MOESM3_ESM.docx]

**Supplementary Figure Legends**

Supplementary Fig. 1: Comparative gene expression of inflamed chondrocytes treated with Dexamethasone (DEX) versus Triamcinolone (TA)

DEX at a concentration of 40nM significantly downregulated the expression of inflammatory mediators interleukin-6 (IL-6, *p=*0.0311) and Matrix Metalloproteinase 3 (MMP-3, *p=*0.0455)) compared to inflamed chondrocytes. DEX at lower (1 and 10nM) and higher (1µM) dosages and TA in all doses (38, 76 and 152µM) failed to ameliorate the effect of the cytokines on inflammatory gene expression.

ns…not significant (*p*≥0.05), *…*p*<0.05, **…*p* <0.01, ***…*p*<0.001

Supplementary Fig. 2: Cell viability (metabolic activity) of chondrocytes after Dexamethasone (DEX) versus Triamcinolone (TA) treatment

Metabolic activity, illustrated as MTT values, of

(A) healthy chondrocytes was not significantly affected by DEX (1nM, 10nM, 40nM and 1 µM),

(B) healthy chondrocytes was significantly increased with TA (T24: 38µM (*p*=0.0358), T48: 76µM (*p*=0.0066)) compared to healthy chondrocytes.

(C) inflamed chondrocytes was not significantly affected by DEX (1nM, 10nM, 40nM and 1 µM) treatment

(D) inflamed chondrocytes was not significantly affected by TA (38µM, 76µM, 152µM) treatment compared to inflamed controls.

ns…not significant (*p*≥0.05), *…*p*<0.05, **…*p* <0.01, ***…*p*<0.001

Supplementary Fig. 3: Proliferation of chondrocytes treated with Dexamethasone (DEX) versus Triamcinolone (TA)

Proliferation (percentage of phase area confluence (%)) of inflamed chondrocytes was not significantly influenced by

(A) DEX treatment (1nM, 10nM, 40nM and 1µM)

(B) TA treatment (38µM, 76µM, 152µM) compared to healthy chondrocytes over the course of 48h.

ns…not significant (*p*≥0.05), *…*p*<0.05, **…*p* <0.01, ***…*p*<0.001

Supplementary Figure 4: Treatment effect of Dexamethasone (DEX: 40nM and 1µM) on healthy and inflamed chondrocytes

(A) DEX treatment (40nM and 1µM) had no significant influence on wound healing of healthy or inflamed chondrocytes.

(B) SA-β-Gal activity significantly increased with culture duration in healthy untreated cells between 24h and 48h (*p*=0.0323), but a single dose of DEX (40nM or 1 µM) did not affect SA-β-Gal activity of healthy or inflamed chondrocytes.

(C) DEX treatment (40nM) of inflamed chondrocytes significantly downregulated inflammatory mediators like interleukin-6 (IL-6: T24 *p*=0.0209, T48 *p*=0.2362) and matrix metalloproteinase -3 (MMP-3: T24 *p*=0.0349, T48 *p*=0.8098) compared to inflamed chondrocytes at T24, but not matrix metalloproteinase-1 (MMP-1: T24 *p=*0.2934, T48 *p=*0.4616). DEX treatment (40nM) of inflamed chondrocytes reduced the expression of collagen type II (Col2: T24 p=0.2625, T48 p=0.6967) and aggrecan (ACAN: T24 *p*=0.6761, T48 *p*=0.4311) compared to inflamed controls. DEX treatment (40nM) of healthy chondrocytes significantly downregulated MMP-1 (*p=*0.0428) and ACAN (*p=*0.0276), and non-significant reduced IL-6 (*p*=0.2159) and COL2 expression (p=0.1943) at T48 compared to healthy controls.

ns…not significant (*p*≥0.05), *…*p*<0.05, **…*p* <0.01, ***…*p*<0.001

Supplementary Figure 5: Venn Diagrams of differentially expressed genes (DEGs) at 24h (T24) and 48h (T48).

Venn diagram of the genes differentially regulated (adjusted *p* < 0.05) between inflamed untreated and healthy (IC-HC), inflamed DEX treated vs inflamed untreated (tDIC-IC) and inflamed DEX treated vs healthy (tDIC-HC) chondrocytes at T24 and T48, overall and divided into up- and down-regulated DEGs.

Supplementary Figure 6: Ingenuity Pathway Analysis of genes differentially expressed genes at 48h

(A) Graphical summary of of the Ingenuity Pathway Analysis (IPA) core analysis for genes differentially expressed between inflamed and healthy chondrocytes at T24.

(B) Graphical summary of the genes differentially regulated between DEX treated and healthy chondrocytes

Genes differentially expressed between inflamed DEX treated vs inflamed untreated chondrocytes at T48 did not yield enough connectable entities with sufficiently high z-scores for a graphical summary.

orange … predicted activation (based on the z-score), blue … predicted inhibition, solid line: direct interaction, dashed line: indirect interaction;

Supplementary Figure 7: Long term implications of Dexamethasone (DEX 40nM and 1µM) treatments.

(A) Metabolic activity, illustrated as MTT values remained unaltered in all comparison groups compared to healthy chondrocytes.

(B) DEX 1µM treatment of healthy but not inflamed chondrocytes led to a significant upregulation of senescence associated gene p53 (*p*=0.0179), but not p21 (*p*=0.7525) or sirt1 (*p*=0.1247), while DEX 40nM treatment of inflamed but not healthy chondrocytes promoted a significant upregulation of sirt1 (*p*=0.0308), but not p53 (*p*=0.5712) or p21 (*p*=0.8231).

ns…not significant (*p*≥0.05), *…*p*<0.05, **…*p* <0.01, ***…*p*<0.001
