# Supplementary figures and images for: Dexamethasone: a double-edged sword in the treatment of osteoarthritis
Source: Sci Rep. 2025 Apr 7;15:11832. doi: 10.1038/s41598-025-96050-2 (PMC11976973; doi:10.1038/s41598-025-96050-2)

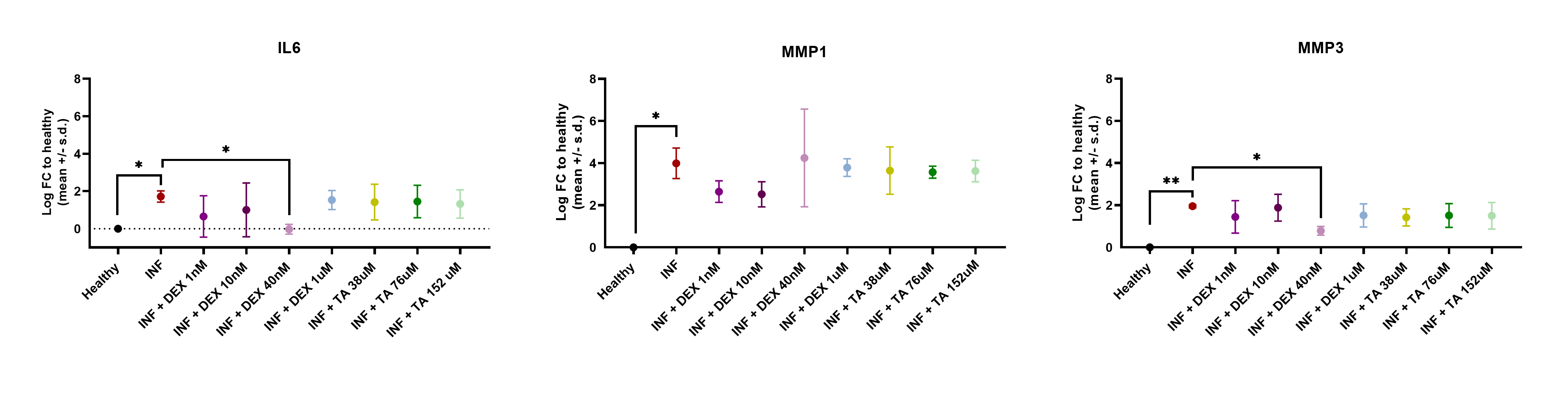

Supplement: Supplementary file 4 — Supplementary Material 4 [file 41598_2025_96050_MOESM4_ESM.tif]

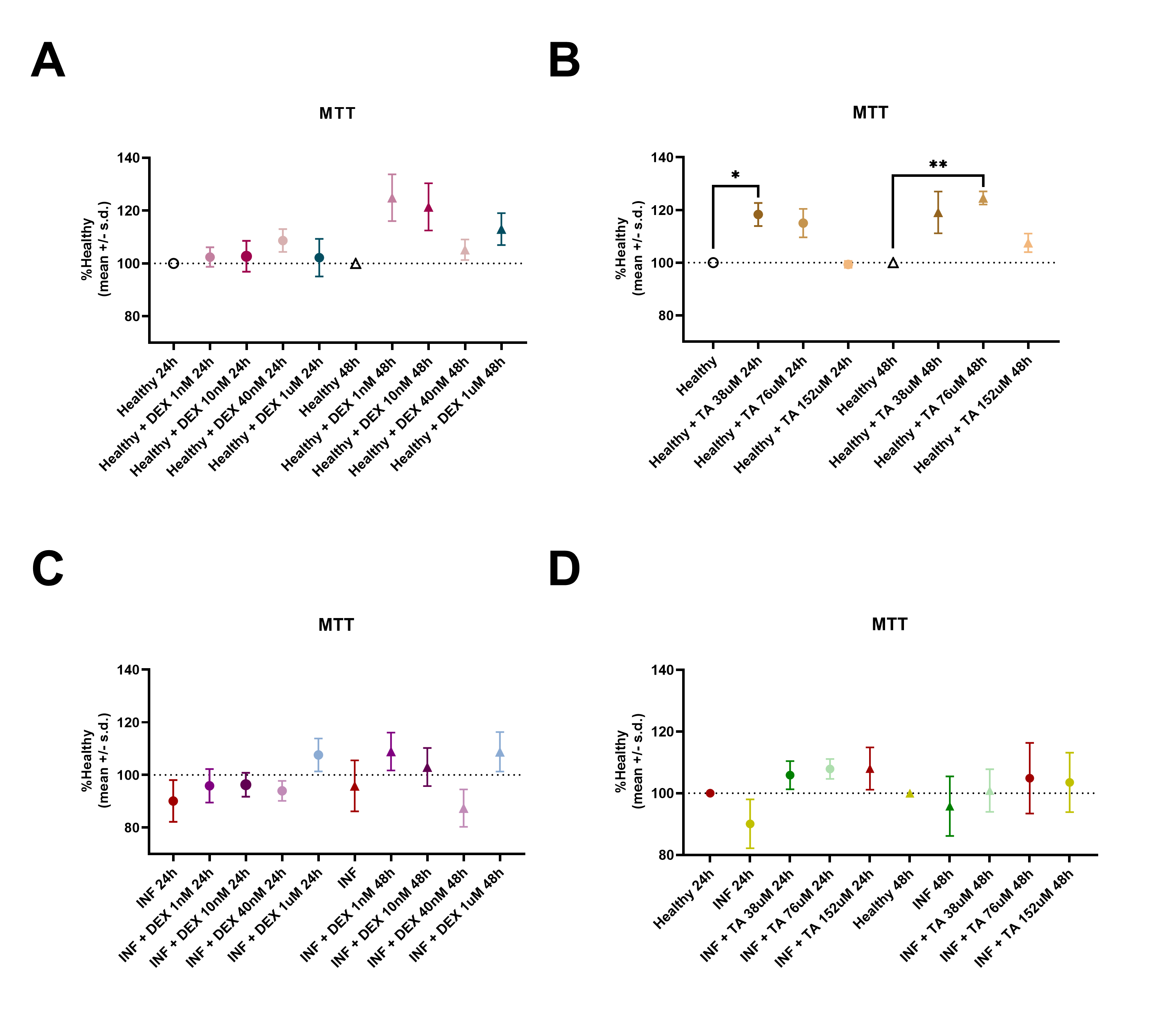

Supplement: Supplementary file 5 — Supplementary Material 5 [file 41598_2025_96050_MOESM5_ESM.tif]

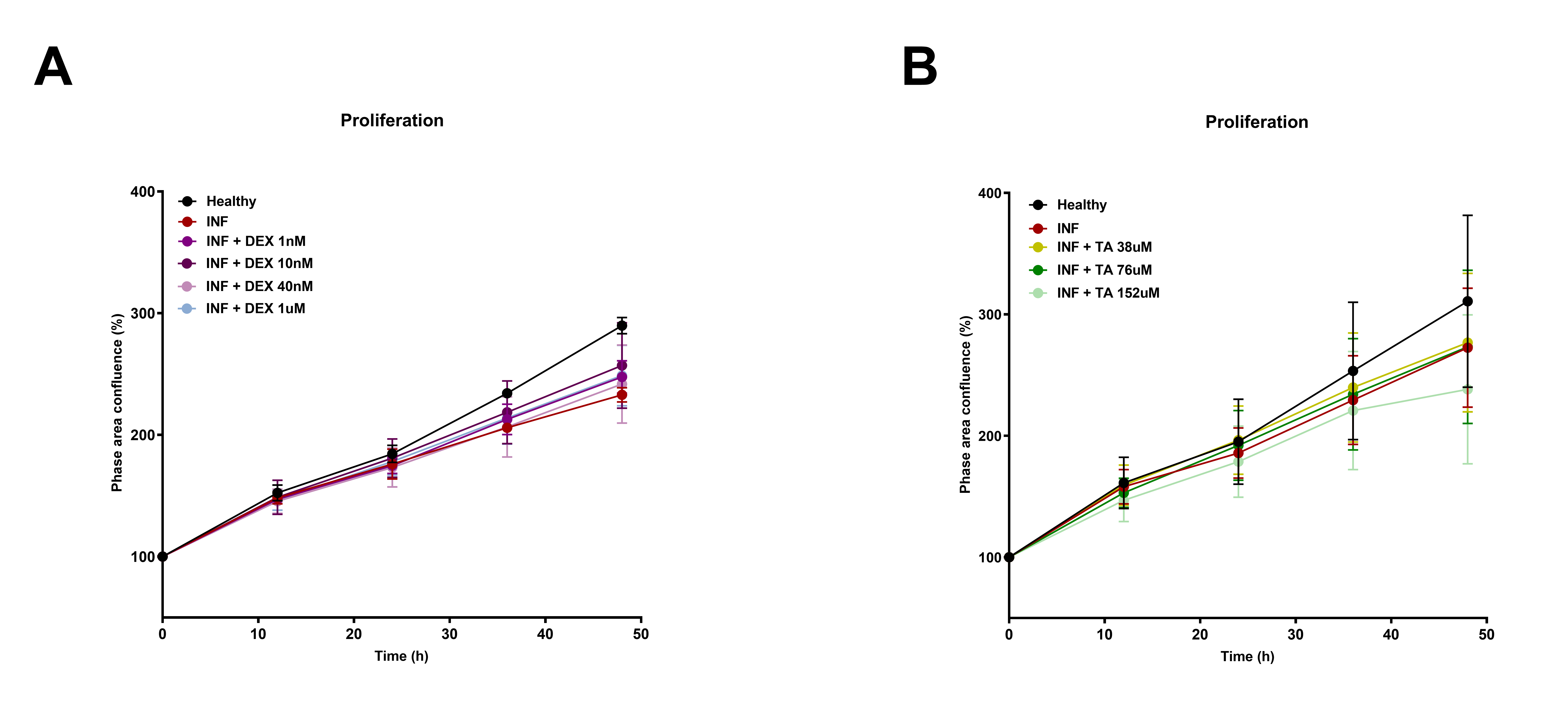

Supplement: Supplementary file 6 — Supplementary Material 6 [file 41598_2025_96050_MOESM6_ESM.tif]

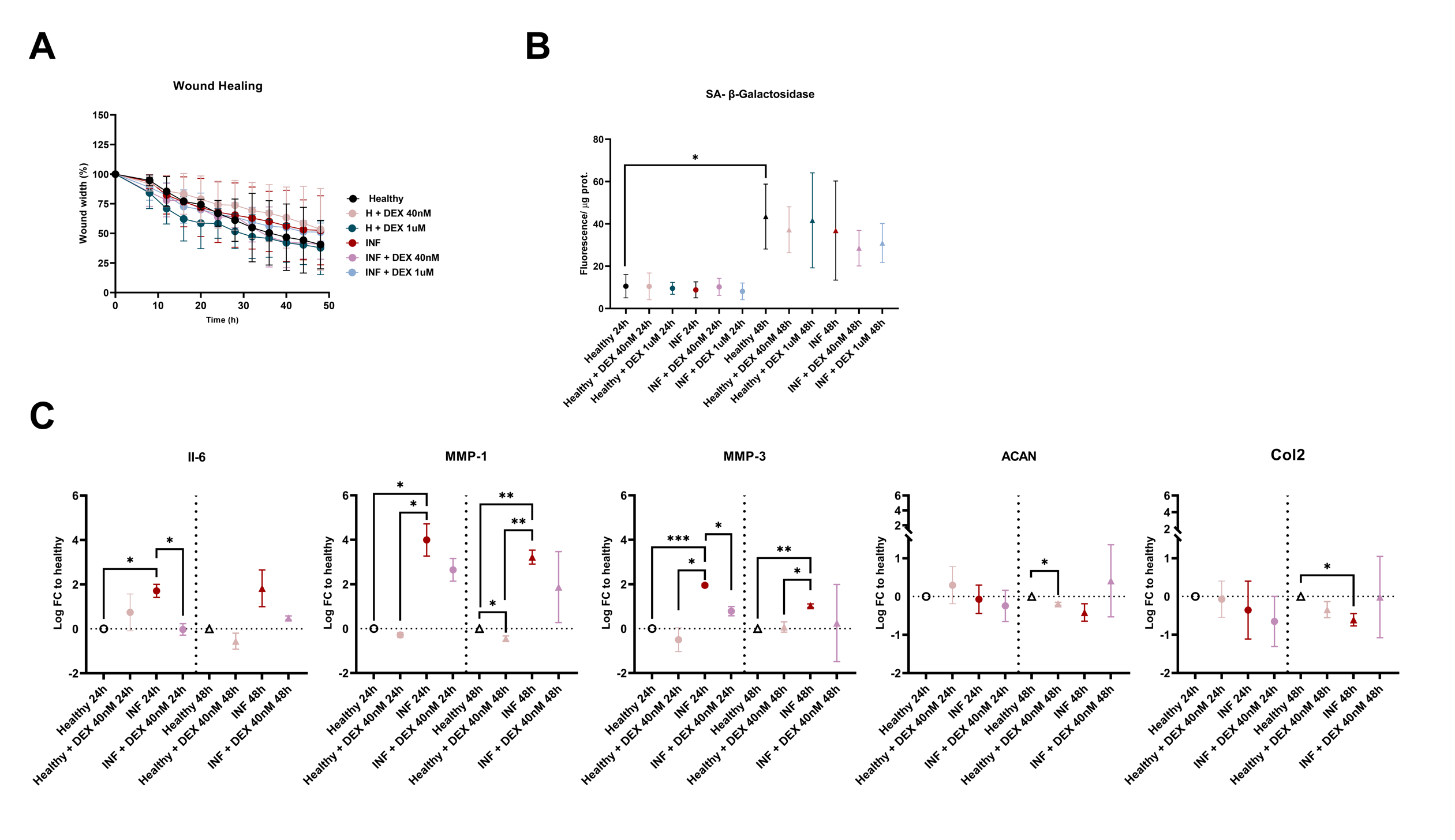

Supplement: Supplementary file 7 — Supplementary Material 7 [file 41598_2025_96050_MOESM7_ESM.tif]

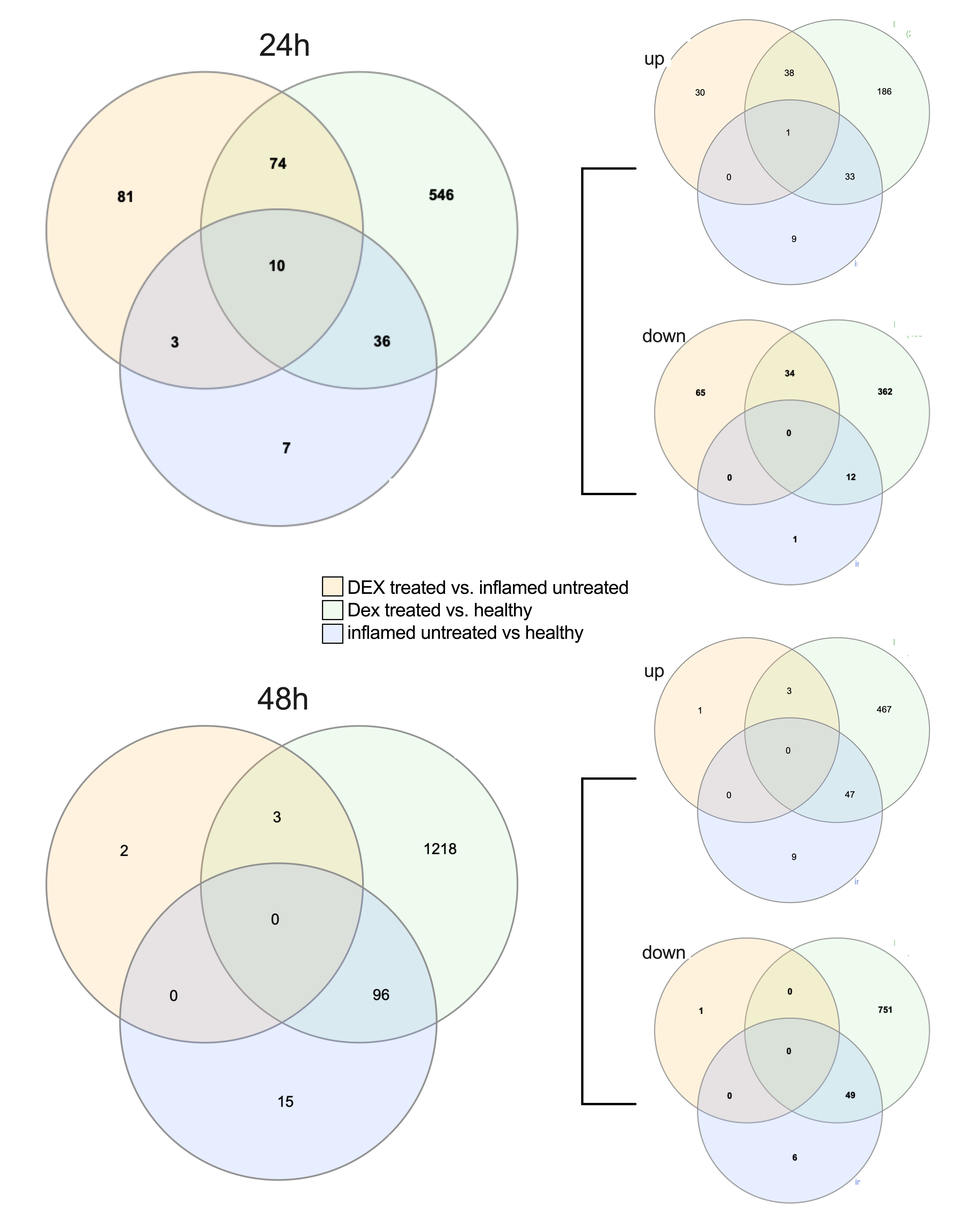

Supplement: Supplementary file 8 — Supplementary Material 8 [file 41598_2025_96050_MOESM8_ESM.tiff]

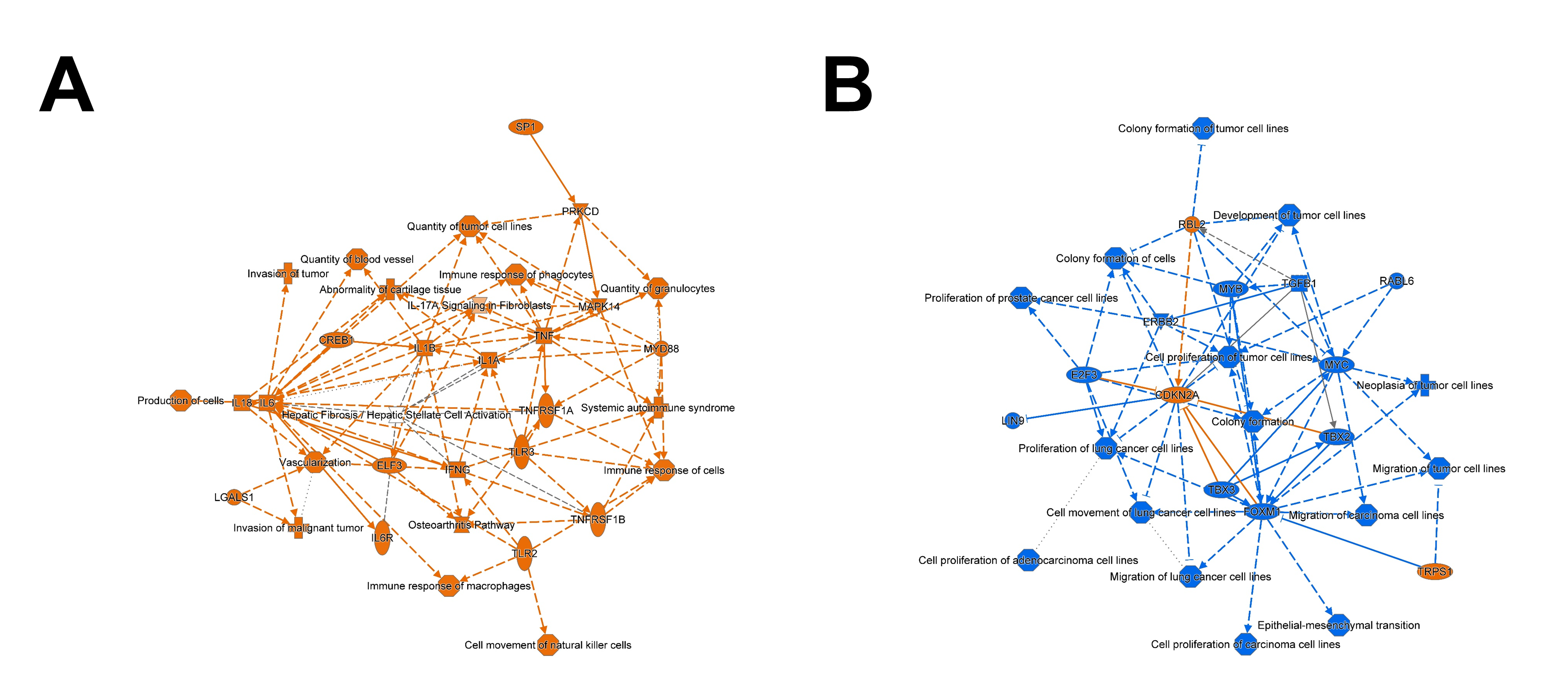

Supplement: Supplementary file 9 — Supplementary Material 9 [file 41598_2025_96050_MOESM9_ESM.tif]

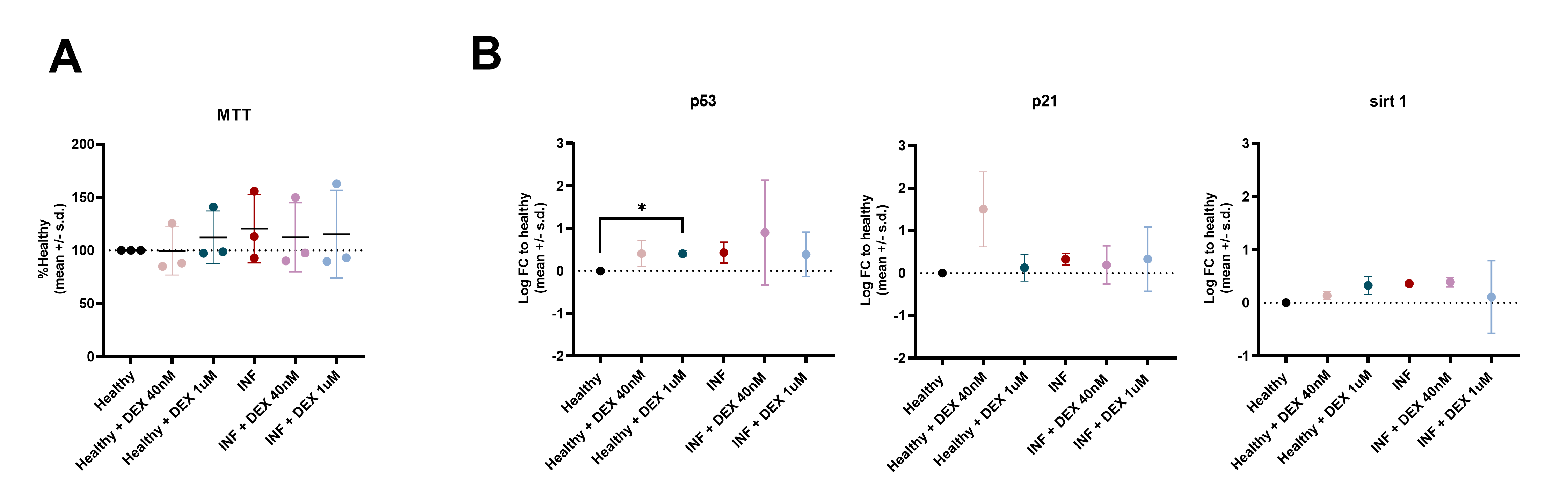

Supplement: Supplementary file 10 — Supplementary Material 10 [file 41598_2025_96050_MOESM10_ESM.tif]
